# The CCL2-CCR2 axis in primary lung cancer and pulmonary metastasis: from molecular mechanisms to therapeutic potentials

**DOI:** 10.3389/fimmu.2026.1871102

**Published:** 2026-09-10

**Authors:** Yujie Huang, Jia Ma, JianPing Bi, Guoliang Pi, Ying Li, Yi Peng, Chuangying Xiao, Xiulin Tuo, Guang Han

**Affiliations:** 1 Department of Radiation Oncology, Hubei Cancer Hospital, Tongji Medical College, Huazhong University of Science and Technology, Wuhan, China; 2 Department of Oncology, Jianghan Oilfield General Hospital, Qianjiang, China; 3 Tongji Hospital, Tongji Medical College, Huazhong University of Science and Technology, Wuhan, China

**Keywords:** CCL2, CCR2, lung cancer, metastasis, NSCLC, TAMs, TME

## Abstract

Lung cancer remains the leading cause of cancer-related mortality worldwide, with metastasis, therapeutic resistance, and an immunosuppressive TME representing major barriers to successful treatment. Among the chemokine signaling networks implicated in these processes, the CCL2–CCR2 axis has emerged as an important regulator of tumor progression. By orchestrating the recruitment of monocytes, tumor-associated macrophages, myeloid-derived suppressor cells, and other stromal components, this pathway can establish an immune-permissive niche that supports angiogenesis, epithelial–mesenchymal transition, extracellular-matrix remodeling, metastatic dissemination, and resistance to chemotherapy, targeted therapies, and immunotherapy. Mechanistically, CCL2–CCR2 signaling interacts with key oncogenic pathways, including PI3K/Akt/mTOR, STAT3, NF-κB, Toll-like receptor signaling, and non-coding RNA-mediated networks, thereby amplifying pro-tumor inflammatory circuits within the lung TME. Although accumulating evidence indicates that the axis can exert context-dependent tumor-suppressive effects under specific biological conditions, such as promoting M1 macrophage recruitment or enhancing antitumor immunity in selected settings, its overall contribution in lung cancer appears predominantly tumor-promoting. This review comprehensively summarizes the molecular mechanisms governing CCL2–CCR2 signaling in primary lung cancer and in lung metastases. We further discuss emerging therapeutic strategies directed at this axis, including CCR2 antagonists, CCL2-neutralizing agents, engineered immune-cell approaches, and rational combinations with immune-checkpoint inhibitors and targeted therapies. Collectively, the current evidence provides a strong biological rationale and preliminary translational support for further investigation of the CCL2–CCR2 axis as a potential therapeutic target and exploratory biomarker in advanced lung cancer. However, clinical validation remains limited, and additional well-designed studies are required to determine whether modulation of this pathway can meaningfully improve patient outcomes.

## Graphical Abstract

**Figure:**
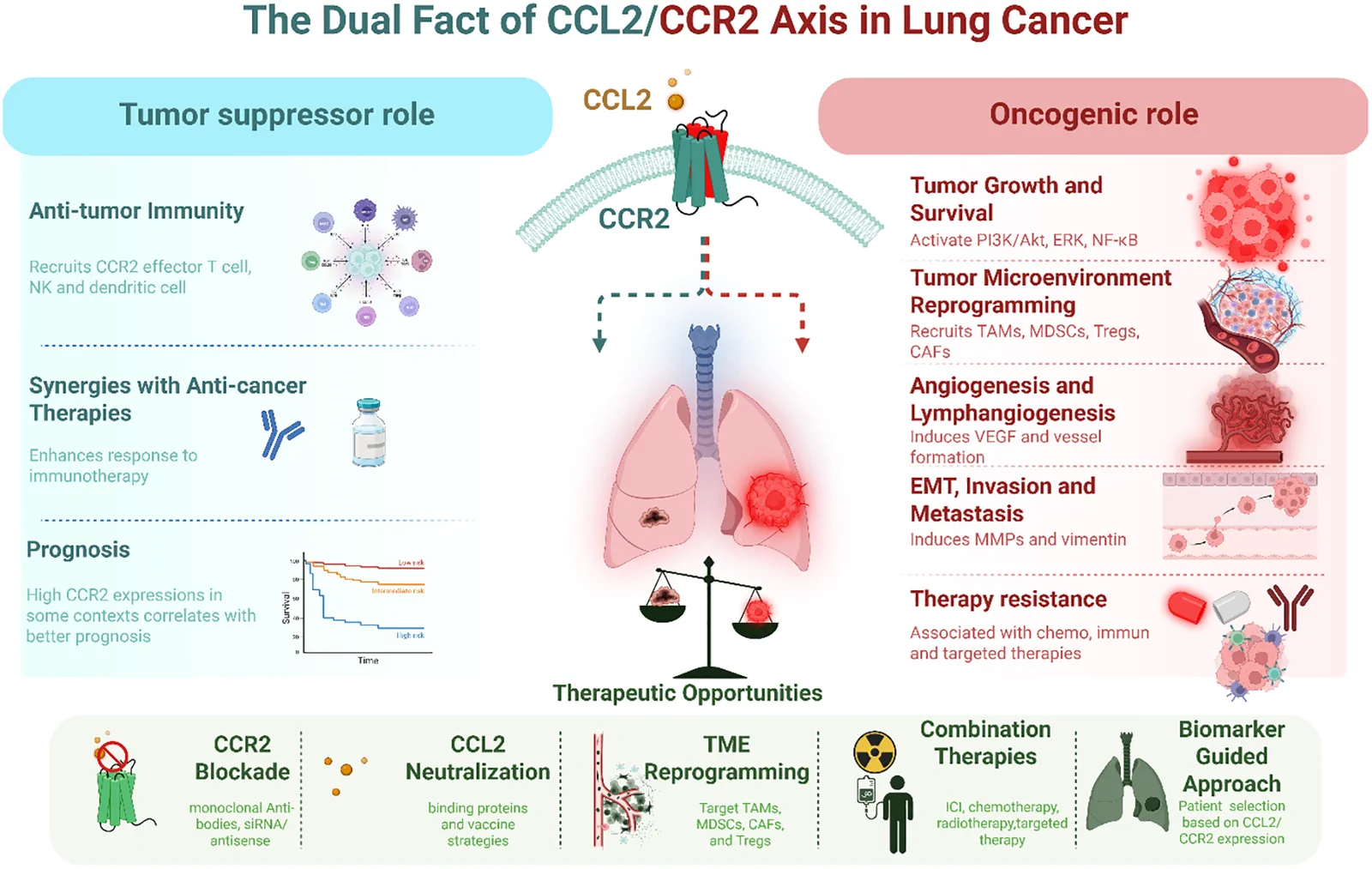

## 1 Introduction

Lung cancer continues to represent the most frequently diagnosed cancer and the foremost cause of cancer-related death worldwide, imposing a major global health burden. The most recent investigations indicate that nearly 2.6 million new cases and 1.9 million deaths occurred in 2024. Non-small cell lung cancer (NSCLC) accounts for the large majority of these diagnoses, comprising approximately 85% of all lung cancer cases (1). Despite advances in targeted therapies and immunotherapy, chemotherapy remains an essential component of treatment across disease stages. However, its clinical utility is often limited by significant toxicity and suboptimal therapeutic efficacy (2). These challenges highlight a critical gap in current management and emphasize the necessity of identifying novel molecular determinants that drive NSCLC progression and therapeutic resistance, thereby paving the way for more precise and effective interventions (3).

In this regard, increasing attention has been directed toward the tumor microenvironment (TME) and its regulatory chemokine networks, among which the CCL2-CCR2 axis has emerged as a pivotal player. CCL2, also known as monocyte chemoattractant protein-1 (MCP-1), is a member of the CC chemokine family that orchestrates immune cell recruitment and inflammatory responses (4). Upon binding to its receptor CCR2, CCL2 mediates the recruitment of monocytes and promotes their differentiation into tumor-associated macrophages (TAMs), which actively reshape the TME (5). Through paracrine signaling, these cells foster immunosuppression, tumor growth, and survival. Importantly, the CCL2-CCR2 axis has also been implicated in the development of drug resistance, as it enhances cancer cell adaptability and sustains a protective niche (6). Moreover, the ability of CCL2 to upregulate its own expression establishes a feed-forward loop that further attenuates the efficacy of chemotherapeutic agents (7).

The central focus of this review is the role of the CCL2-CCR2 axis in primary lung cancer progression, metastasis, and therapeutic resistance. Building on this mechanistic foundation, accumulating evidence highlights the context-dependent role of the CCL2-CCR2 axis in lung cancer progression and metastasis (8). While it predominantly drives tumor progression, the axis also displays tumor-restraining potential in specific settings for instance, via M1 macrophage recruitment or in SCLC, where CCL2 silencing promotes growth. As detailed in the mechanistic insights of this study, factors such as platelet-derived growth factor-BB (PDGF-BB) activate MAPK and PI3K/Akt signaling pathways to induce CCL2 expression in lung cancer cells. This, in turn, enhances CCR2-dependent macrophage recruitment, establishing a reciprocal interaction between tumor cells and TAMs that drives invasive behavior (9). Similarly, estrogen receptor α (ERα) has been shown to transcriptionally activate CCL2, leading to increased macrophage infiltration, M2 polarization, and matrix metalloproteinase production, all of which contribute to extracellular matrix remodeling and metastatic dissemination (10). These processes are further amplified by feedback loops involving pathways such as CXCL12/CXCR4, underscoring the dynamic and self-reinforcing nature of this chemokine axis in NSCLC.

While the primary emphasis remains on primary lung cancer, we also discuss the CCL2-CCR2 axis in the context of lung metastases originating from other common malignancies, breast, colorectal, melanoma, osteosarcoma, and renal cell carcinoma. This integrated approach is justified because the lung is a frequent metastatic site, and the axis plays conserved roles in pre-metastatic niche formation, myeloid cell recruitment, and colonization within the pulmonary microenvironment. Understanding these overlapping mechanisms provides a more comprehensive view of CCL2-CCR2 biology and highlights shared therapeutic vulnerabilities relevant to patients with advanced, metastatic disease. Given these interconnected mechanisms, a comprehensive understanding of the CCL2-CCR2 axis is essential for advancing lung cancer research and therapy. Therefore, this review aims to systematically elucidate the molecular underpinnings of the CCL2-CCR2 signaling network in lung cancer and metastasis, with particular emphasis on its dual functional roles within the TME. We further explore its interactions with key oncogenic pathways, its impact on immune modulation and therapeutic resistance, and the emerging strategies designed to therapeutically target this axis. By integrating current molecular insights with translational perspectives, this article provides a cohesive framework to inform future investigations and support the development of innovative, mechanism-based therapeutic approaches for lung cancer.

## 2 The molecular mechanism of CCL2-CCR2 chemokine axis in lung cancer

The CCL2–CCR2 axis functions as a central signaling hub in the lung-cancer TME. Across diverse upstream inputs, engagement of this axis consistently drives a core set of downstream processes: CCR2-dependent recruitment of circulating monocytes, their differentiation into M2-polarized TAMs and monocytic myeloid-derived suppressor cells (MDSCs), suppression of cytotoxic T-cell and NK-cell activity, promotion of epithelial–mesenchymal transition (EMT), extracellular-matrix remodeling, angiogenesis, and facilitation of local invasion and distant metastasis (11). These shared consequences are described in detail only when uniquely modulated by a particular regulator; otherwise they are referred to collectively.

### 2.1 M2 polarization and TAMs interaction with CCL2-CCR2

Macrophages in the lung cancer TME exhibit high plasticity, polarizing into pro-inflammatory M1 or pro-tumorigenic M2 phenotypes depending on local signals. M1 macrophages, induced by IFN-γ and LPS, produce TNF-α, IL-6, iNOS, and major histocompatibility complex (MHC)-II, exerting anti-tumor effects through cytotoxicity, phagocytosis, and effector T/NK cell activation (12). Conversely, M2-like TAMs, driven by IL-4/IL-13 and tumor-derived factors such as CCL2, express CD163, CD206, and Arg1 while secreting IL-10, TGF-β, and VEGF; they promote tumor progression by enhancing angiogenesis, extracellular matrix remodeling via matrix metalloproteinases (MMPs), EMT, invasion, metastasis, and immunosuppression. In lung cancer, predominant M2 polarization creates a permissive niche that sustains chronic inflammation and immune evasion, underscoring the central importance of the CCL2-CCR2 axis in this skewing (13). TAMs constitute a major immunosuppressive component of the lung cancer microenvironment, with M2-polarized subsets predominating and actively shaping chemokine networks. These M2 TAMs sustain tumor progression by secreting pro-angiogenic and anti-inflammatory factors while simultaneously responding to and amplifying chemokine gradients, thereby reinforcing recruitment of additional MDSCs and creating a self-perpetuating immunosuppressive niche (14). In lung cancer, this regulatory function is particularly evident in the context of CCL2, where M2 TAMs both produce and migrate toward this chemokine, linking local polarization events to broader myeloid infiltration and immune evasion (15).

The CCL2-CCR2 axis serves as a central driver of TAM recruitment and M2 polarization in lung cancer. TAMs largely originate from CCR2-expressing monocytes mobilized from the bone marrow by tumor-derived CCL2, a process that not only increases TAM density but also favors their differentiation toward the pro-tumor M2 phenotype characterized by elevated expression of CD163, CD206, Arg1, and S100A8/A9. Multiple tumor-intrinsic regulators converge on this axis (16). Multiple tumor-intrinsic regulators converge on this axis. PRPS2 positively correlates with CCL2; its knockout lowers CCL2 secretion, reduces TAM/MDSC infiltration and elevates CD4^+^/CD8^+^ T-cell percentages effects fully reversed by CCL2 neutralization or myeloid depletion (17). Parallel mechanisms reinforce this axis through transcriptional and post-translational control of CCL2. In NSCLC, elevated CtBP1 activates the nuclear factor-κB (NF-κB) pathway by promoting p65 nuclear translocation, directly transactivating CCL2 and enhancing both TAM recruitment and M2 polarization. Clinical NSCLC specimens show a strong positive correlation between CtBP1 levels and CD163^+^ TAM density, while orthotopic mouse models confirm that CCR2 antagonism abolishes CtBP1-mediated TAM accumulation and tumor progression (18). Similarly, the neddylation pathway sustains CCL2 transactivation by enabling Cullin-RING ligase activity that degrades IκBα, thereby derepressing NF-κB; pharmacological or genetic inactivation of neddylation stabilizes IκBα, suppresses CCL2 production, diminishes monocyte chemotaxis, and reduces TAM infiltration, an effect rescued by exogenous CCL2. Patient data further link high NEDD8 expression to elevated CCL2 and inferior survival in lung adenocarcinoma, illustrating a clinically relevant feed-forward loop (19).

Additional tumor-cell-autonomous signaling amplifies CCL2-driven TAM engagement. FCRLB was identified as a key prognostic regulator in NSCLC through multi-omics and ensemble machine-learning analyses. High FCRLB expression in tumor cells activates PI3K and ROS pathways, drives CCL2 production, and promotes M2 macrophage polarization, thereby enhancing lung cancer progression. shRNA knockdown and murine xenograft models validated its pro-tumor role via ROS/PI3K-mediated cytokine secretion in the immunosuppressive TME (20). PDGF-BB acting through PDGF-Rβ in lung cancer cells triggers an autocrine loop that upregulates MCP-1/CCL2 via sequential activation of mitogen-activated protein kinase (MAPK) and PI3K/Akt pathways, culminating in NF-κB-dependent transcription. Conditioned medium from PDGF-BB-stimulated lung cancer cells robustly recruits macrophages in a CCL2- and CCR2-dependent manner; blocking antibodies against MCP-1 abolish this chemotaxis. Recruited TAMs, in turn, feedback to cancer cells by inducing MMPs and VEGF expression, thereby enhancing extracellular-matrix degradation and metastatic potential and closing a vicious paracrine circuit centered on the CCL2-CCR2 axis (9). Non-tumor stromal elements further modulate M2 polarization through the same chemokine network (21). Schwann cells within the peripheral nervous system component of the lung TME secrete high levels of CCL2, CXCL5, CXCL12, and CXCL8, promoting M2 polarization of peripheral-blood-monocyte-derived macrophages in a contact-independent fashion. This Schwann-cell-driven M2 shift enhances lung cancer cell proliferation, establishing a bidirectional “Schwann cell–M2 macrophage–cancer cell” axis that integrates neural and immune compartments to sustain tumor growth (22). Counter-regulatory mechanisms exist: Nrf2 activation selectively repolarizes tumor-educated macrophages toward an M1 phenotype, down-regulating CCL2 and restoring anti-tumor activity in a strictly Nrf2-dependent fashion (23).

In conclusion, while M2 polarization and TAMs interact with the CCL2-CCR2 axis as predominant sensors and amplifiers of immunosuppressive signaling in lung cancer, counter-regulatory mechanisms reveal its duality. For example, Nrf2 activation repolarizes TAMs toward M1 phenotypes with reduced CCL2, and in SCLC epigenetic restoration of CCL2 enhances antitumor macrophage infiltration. This positions the axis as a convergent yet context-sensitive vulnerability.

### 2.2 CCL2-CCR2 in recruitment and function of monocytes

Monocytes play a pivotal role in shaping the TME across multiple cancers, where the CCL2-CCR2 axis serves as a primary chemotactic pathway that recruits circulating monocytes, promotes their differentiation into tumor-associated macrophages or myeloid-derived suppressor cells, and orchestrates pro-tumor functions including immunosuppression, extracellular matrix remodeling, and metastatic support (24, 25).

In lung squamous cell carcinoma, integrative multi-omics analyses have pinpointed an inflammatory monocyte-rich subset defined by elevated CD14 expression that associates more strongly with reduced patient survival than tumor-associated macrophages or activated dendritic cells. Tumor cells in metastatic subclones secrete elevated TNFα, which activates NFκB signaling to drive CCL2 production; this chemokine then engages CCR2 on monocytes, establishing a feed-forward loop that amplifies recruitment because both recruited inflammatory monocytes and their macrophage progeny further release TNFα (26). Parallel investigations in non-small cell lung cancer cell models reveal that thromboxane A2 signaling through its TP receptor induces CCL2 expression via an SP1-dependent mechanism that requires Hsp90 modulation but is independent of AP-1, PKC, or ERK pathways. The resulting CCL2 gradient recruits monocytes and macrophages, which in turn stimulate lung cancer cells to upregulate matrix metalloproteinases and vascular endothelial growth factor, creating a positive feedback circuit that heightens invasive potential irrespective of heterogeneous TP levels within tumor subpopulations (27).

Patient-derived data reinforce these dynamics: peripheral blood mononuclear cells from individuals with non-small cell lung cancer release markedly higher CCL2 levels upon LPS stimulation compared with those from healthy smokers or non-smokers, while immunohistochemical studies show tumor-cell MCP-1 expression correlating with macrophage infiltration; although prognostic implications vary by macrophage polarization state, with M2-like phenotypes linked to lymph-node metastasis and poorer outcomes, positive MCP-1 staining in tumors has been associated with smaller lesion size and, in some cohorts, improved survival, potentially reflecting enhanced recruitment of tumoricidal monocytes or natural killer cells (28). Recruited inflammatory monocytes additionally express high levels of Factor XIII A, which catalyzes rapid fibrin cross-linking to generate a provisional matrix scaffold that facilitates cancer-cell invasion, mirroring the “non-healing wound” paradigm of solid tumors; these cells may overlap phenotypically with monocytic myeloid-derived suppressor cells yet exhibit distinct functions in the lung cancer milieu, as they show no enrichment for iNOS or arginase-1 and instead drive metastasis through structural and microenvironmental remodeling (29). Therefore, the CCL2-CCR2 axis emerges as a central regulator of monocyte recruitment and pathogenic function in lung cancer, underscoring its value as a context-specific vulnerability amenable to targeted blockade (**Table 1**).

**Table 1:** Key upstream regulators of CCL2-CCR2 activity and their impact on lung cancer biology.

Upstream regulator	Role of CCL2	CCL2-CCR2 activity	Type of study	Cancer and cell lines	Axis	Upstream regulator effects	Ref
Galectin-3	Oncogenic/immunosuppressive	↑ CCL2-CCR2	*In vitro* and *in vivo*	The human lung cancer cell line (A549, and H292), mouse lung adenocarcinoma cell line (LLC), mouse mononuclear macrophage leukemia cell line (RAW264.7), and human embryonic kidney epithelial cell line (293T)	Galectin-3/CCL2-CCR2/TREM2	• Disrupted TREM2-driven phagocytosis • Drove TREM2-positive macrophages toward an immunosuppressive TAM phenotype	(64)
Engineered CCR2 (co-expressed with WT1-specific TCR in CD8^+^ T cells)	Tumor-derived chemoattractant/enhancer of T-cell responsiveness	↑ CCL2-CCR2	*In vitro* and *in vivo*	Human small-cell lung cancer cell line (LK79), other lung cancer lines (LC99A, LC11-18)	CCL2-CCR2/WT1-specific TCR	• Enabled efficient CCL2-driven tumor trafficking • boosted *in vivo* anti-lung cancer efficacy • CCL2 further enhanced WT1-specific TCR signaling, increasing IFN-γ production and T-cell responsiveness.	(65)
Waterpipe smoke condensate (WPSC)	Pro-inflammatory/EMT/stemness promoter	↑ CCL2	*In vitro*	Human NSCLC cell lines (A549 and H460)	WPSC/CASP1, IL1B, IL6 and CCL2	• Induced CCL2, IL-1β, IL-6 and CASP1 expression • Promoted EMT increased DNA damage, senescence • Reduced NK cell-mediated killing of A549 cells	(66)
Cadmium (Cd) via FAP upregulation in fibroblasts	Pro-tumorigenic	↑ CCL2	*In vitro*	Human lung fibroblasts (MRC-5); NSCLC lines (A549, H460)	Cd/FAP/iCAFs-like/CCL2, and IL-6	• Promoted proliferation, migration, and invasion of lung cancer cells	(67)
CTNNAL1 (catenin alpha-like 1)	Pro-tumorigenic	↑ CCL2	*In vitro*	Human lung cancer cell lines (A549, NCI-H460)	CTNNAL1/CCL2/CSC/EMT	• Promoted CSC stemness, EMT, migration/invasion, and radioresistance	(68)
KCNAB2	Tumor-derived chemokine for immune cell recruitment	↑ CCL2	*In vitro*	LUAD (A549, H23 cell lines)	KCNAB2/CCL2, CCL3, CCL4, CCL18, CXCL9, CXCL10, and CXCL12/immune infiltration	• KCNAB2 overexpression upregulates multiple chemokines (including CCL2) critical for recruitment of anti-tumor immune cell.	(69)
Anti-CCL2/CCL12 mAbs	Protumorigenic/immunosuppressive	↓ CCL2-CCR2	*In vitro* and *in vivo*	Murine NSCLC (TC1, LLC, LKR, LKR-M), mesothelioma (AB12, AE17)	CCL2-CCR2/TAM polarization/CD8^+^ T cells	• Inhibited primary tumor growth • Reduced spontaneous lung metastases	(70)
FSCN1	Pro-tumorigenic	↑ CCL2	*In vitro* and *in vivo*	Human LUAD cell lines (A549)	FSCN1/IGF1R/M-CSF, IL4/PTPRF/M2 polarization/CCL2, IGF1, IL10	• FSCN1 activates PI3K-AKT and JAK-STAT, promotes M-CSF/IL4 secretion · IL4/M-CSF upregulate PTPRF in macrophages • Enhanced glycolysis and M2 polarization · M2 macrophages secrete CCL2	(71)
Caveolin-1 (Cav-1)	Pro-metastatic	↑ CCL2	*In vitro* and *in vivo*	LUAD (A549, H23 cell lines); Human BC cell lines (MDA-MB-231 and MCF-7); human lung fibroblasts (HFL-1); and lung macrophages (THP-1)	PTEN/CCL2/VEGF-A	• Facilitated M2 macrophage polarization & angiogenesis · • Promotes BC lung metastasis	(72)
Endothelial CCR2	Immunosuppressive/pro-metastatic	↑ CCL2-CCR2	*In vitro* and *in vivo*	Mouse lung carcinoma (LLC1.1), mouse colon carcinoma (MC-38GFP); primary lung endothelial cells; bEnd.3 endothelial cell line	CCL2-CCR2/endothelial cytoskeleton (pMLC2, VE-cadherin/β-catenin)	• CCL2 stimulated endothelial MLC2 phosphorylation, cell retraction, and junction loosening	(73)

### 2.3 CCL2-CCR2 in recruitment and function of MDSCs

The CCL2-CCR2 axis plays a critical role in cancers by facilitating monocyte recruitment and modulating MDSC functions to foster an immunosuppressive environment that drives tumor progression and metastasis (6). MDSCs are heterogeneous immature myeloid cells that accumulate in tumors and systemically in cancer patients. They exert potent immunosuppression primarily through arginase-1, iNOS, ROS, and cytokines such as TGF-β and IL-10. In lung cancer, especially NSCLC, both monocytic MDSCs and polymorphonuclear MDSCs expand markedly, correlating with advanced stage, poor prognosis, and therapy resistance. These cells suppress CD8^+^ T-cell function, expand regulatory T cells, and promote angiogenesis and EMT, fostering tumor growth and metastasis (30, 31). Tumor-derived CCL2 directly recruits CCR2^+^ monocytic precursors via PI3K/Akt and JAK/STAT signaling, establishing a self-reinforcing loop especially prominent in KRAS-mutant and EMT-enriched tumors (32, 33).

In contrast, indirect mechanisms further amplify MDSC activity within the lung TME. For instance, EMT-positive metastatic lung adenocarcinoma cells secrete elevated CXCL1, CXCL5, and CCL2, which recruit Hdc^+^ PMN-MDSCs primarily via upregulated CXCR2. While CCL2 itself can influence PMN-MDSC migration to a lesser extent, the recruited Hdc^+^ cells secrete increased TGF-β1 that induces β-catenin translocation in cancer cells, thereby promoting EMT through paracrine signaling. Ablation of these MDSCs or their TGF-β1 significantly reduces tumor burden and EMT rates, consistent with the poorer prognosis observed in EMT^+^ cases (34). Pharmacologic or genetic CCR2 blockade attenuates MDSC influx, restores T-cell infiltration and synergizes with checkpoint inhibitors by reducing arginase and ROS-mediated suppression. EphA2-driven chemokine production supplies additional arginase and TGF-β, reinforcing CD8^+^ T-cell exclusion (29).

Pharmacologic or genetic blockade of CCR2 effectively attenuates this CCL2-driven MDSC influx, restores T-cell infiltration, and synergizes with checkpoint inhibitors by reducing arginase activity and ROS-mediated T-cell suppression. Overall, the interplay of direct chemotactic recruitment and indirect microenvironmental amplification by the CCL2-CCR2 axis in monocyte and MDSC dynamics offers valuable molecular insights and therapeutic avenues for combating lung cancer metastasis.

### 2.4 Crosstalk with cancer-associated fibroblasts and stromal components

CAFs serve as key orchestrators of chemokine regulation within the TME of lung cancer, dynamically modulating the secretion of soluble factors that shape immune cell trafficking, stromal remodeling, and tumor progression (35). Through paracrine signaling and direct cellular interactions, CAFs integrate cues from cancer cells, immune infiltrates, and the surrounding stroma to establish a supportive niche that sustains malignancy (36).

CAFs act as key chemokine hubs. In lung squamous-cell carcinoma, CAFs secrete high levels of CCL2 that recruit CD14^+^CCR2^+^ monocytes and reprogram them into IDO1^+^/NOX2^+^ monocytic MDSCs, generating ROS that suppress CD8^+^ T cells; this interaction is more pronounced than in adenocarcinoma and is linked to smoking-related fibrosis (37, 38). Reprogramming of normal fibroblasts (NFs) into tumor-promoting CAFs further amplifies CCL2-CCR2 signaling. Downregulation of miR-1 and miR-206, together with miR-31 upregulation, drives CAF differentiation and markedly increases secretion of CCL2 and VEGFA. These miRNA-regulated CAFs enhance cancer cell migration, colony formation, angiogenesis, and TAM recruitment via CCL2–CCR2 signaling. Functional studies confirmed CCL2/VEGFA as key mediators, with miR-1 and miR-206 directly targeting their 3’-UTRs, while miR-31 suppresses the tumor suppressor FOXO3a, further derepressing VEGFA without causing significant fibroblast apoptosis. This miRNA network thus sustains a CCL2-overproducing CAF state that promotes sustained CCR2-dependent myeloid infiltration and metastasis (39).

Inflammatory cues from the adaptive immune compartment further tune CAF secretory profiles toward enhanced CCL2-CCR2 activity. Effector cytokines IFNγ and TNFα, released by tumor-infiltrating TH1 lymphocytes, induce patient-derived NSCLC CAFs to adopt an inflammatory CAF (iCAF) phenotype characterized by robust upregulation of CCL2, alongside CCL3, CCL4, CXCL8, and CCL20. This cytokine-driven shift promotes myeloid cell and regulatory T-cell recruitment while simultaneously suppressing CXCL12, a chemokine that otherwise sequesters T cells in stromal compartments and recruits additional suppressive populations. The resulting secretome favors a dynamic balance: although TH1-attracting chemokines (CXCL9, CXCL10, CXCL11) are also elevated, the dominant CCL2 output reinforces CCR2-mediated monocyte influx and MDSC generation (40). Complementary studies demonstrate that Pharmacologic agents such as echinacoside attenuate CAF activation and NF-κB/STAT3/Akt-dependent CCL2 transcription (41).

In summary, CAFs function as central hubs in the CCL2-CCR2 axis within the lung cancer TME, integrating miRNA reprogramming, inflammatory cytokine signals, and direct stromal-immune crosstalk to recruit and educate immunosuppressive myeloid cells while fostering tumor progression. This multifaceted interaction not only explains subtype-specific immune landscapes in NSCLC but also unveils synergistic therapeutic opportunities, ranging from CCR2 antagonists and dual CCL2/VEGFA blockade to miRNA restoration and ROS-scavenging strategies, that hold promise for dismantling CAF-driven immunosuppression and improving clinical outcomes (**Table 1**).

### 2.5 Toll-like receptors mediated regulation of CCL2-CCR2 axis

**Figure 1:**
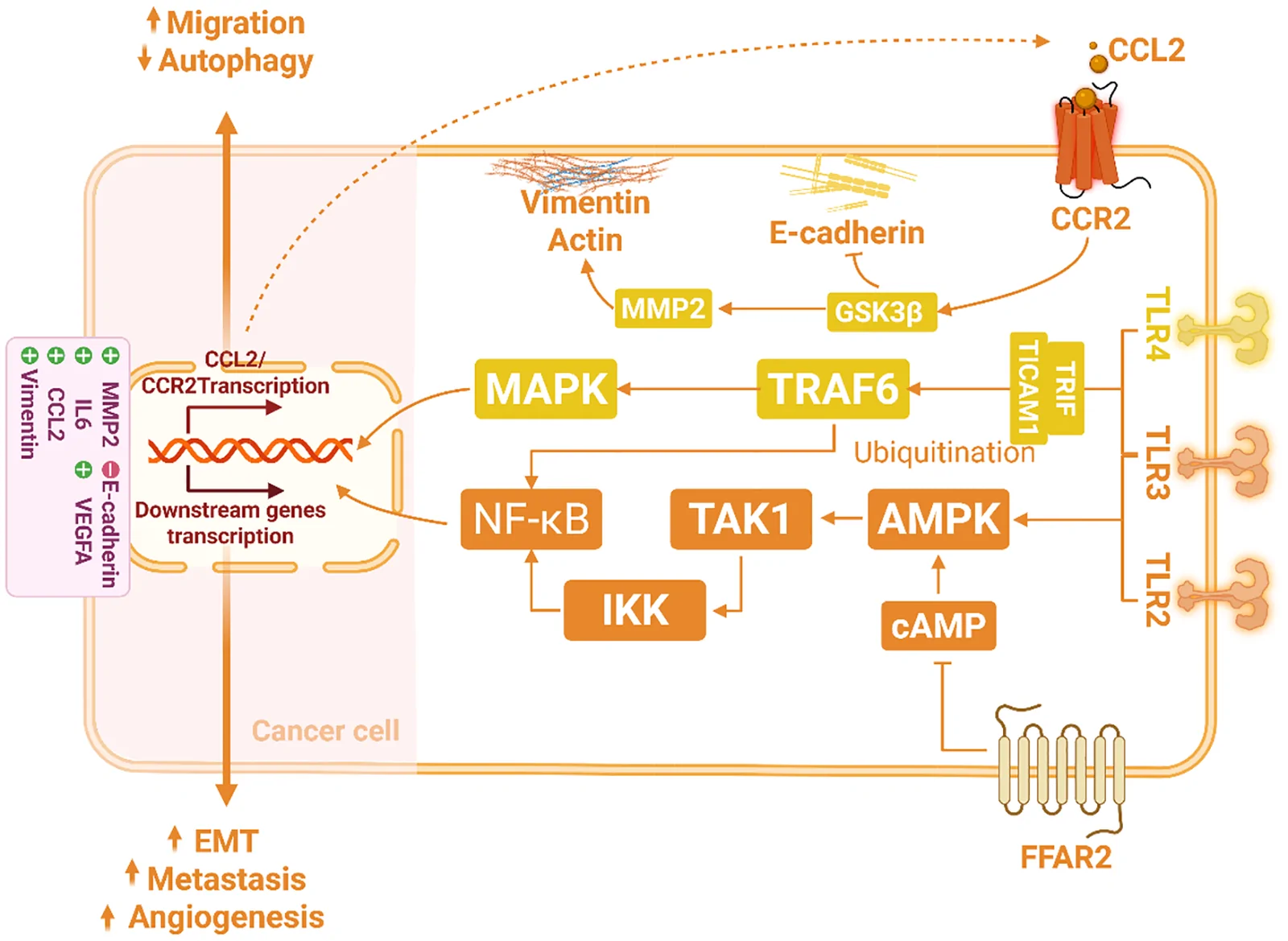
TLR-driven regulation of the CCL2–CCR2 axis in lung cancer progression. Schematic representation of how Toll-like receptors (TLR2, TLR3, and TLR4) orchestrate activation of the CCL2–CCR2 signaling axis in lung cancer cells. Engagement of TLRs stimulates downstream signaling through cAMP/AMPK/TAK1, leading to activation of NF-κB and MAPK pathways, which drive transcriptional upregulation of CCL2 and other pro-tumorigenic mediators, including IL-6, CCL20, VEGFA, and MMP2. Secreted CCL2 reinforces autocrine and paracrine signaling via CCR2, promoting cytoskeletal remodeling, EMT, and enhanced migratory and invasive capacities. TLR3 and TLR4 additionally sustain signaling through TICAM1-dependent autophagy and K63-linked ubiquitination of TRAF6, establishing a positive feedback loop that prolongs NF-κB/MAPK activation. FFAR2 acts as a negative regulator of this axis by suppressing cAMP and downstream AMPK–TAK1 signaling, whereas reduced microbiome-derived short-chain fatty acids diminish this inhibitory effect. Collectively, these interconnected pathways converge to enhance tumor cell migration, metastasis, angiogenesis, and inflammatory remodeling of the TME.

Toll-like receptors (TLRs) function as key innate immune sensors that critically regulate chemokine production in lung cancer, thereby activating the CCL2-CCR2 axis to drive tumor cell migration, invasion, and metastasis (42). Activation of TLR2, TLR3, and TLR4 in NSCLC cells triggers robust secretion of CCL2 along with IL-6, CCL20, vascular endothelial growth factor (VEGF), and MMP2, amplifying autocrine and paracrine signaling through CCR2 that sustains a pro-tumorigenic inflammatory microenvironment (**Figure 1**).

TLR2- and TLR3-mediated signaling potently engages the CCL2-CCR2 axis via NF-κB activation through the cyclic adenosine monophosphate (cAMP)/AMP-activated protein kinase (AMPK)/transforming growth factor-β-activated kinase 1 (TAK1) pathway. Clinical TCGA analyses and an NSCLC patient cohort (n=42) demonstrated a strong inverse correlation between Free fatty acid receptor 2 (FFAR2) and TLR2/TLR3 expression. Gene set enrichment analysis further revealed marked enrichment of cancer modules, innate signaling, and cytokine-chemokine gene sets in tumors exhibiting low FFAR2 and high TLR2/TLR3. Functionally, TLR2 or TLR3 stimulation enhanced migration, invasion, and colony formation in human lung cancer cells by elevating CCL2, IL-6, and MMP2 production. The short-chain fatty acid propionate, acting as an FFAR2 agonist, counteracted these effects by suppressing cAMP levels and attenuating AMPK-TAK1-NF-κB activation; conversely, FFAR2 knockout in A549 and H1299 cells dramatically amplified TLR2/TLR3-driven responses, confirming direct antagonism of the CCL2-CCR2 axis. Reduced lung microbiome diversity in cancer patients limits propionate availability, thereby derepressing TLR2/TLR3 signaling (43). TLR3- and TLR4-mediated regulation similarly amplifies the CCL2-CCR2 axis through TIR domain-containing adapter-inducing interferon-β (TICAM1)-dependent autophagy. This process maintains K63-linked ubiquitination of TNF receptor-associated factor 6 (TRAF6), enabling sustained MAPK and NF-κB activation and establishing a positive feedback loop with autophagy components such as ATG7. Consequently, TLR3/TLR4 stimulation markedly increases CCL2, CCL20, IL-6, VEGFA, and MMP2 secretion, directly promoting lung cancer cell motility and invasion (44). Altogether, TLR2-, TLR3-, and TLR4-driven activation of the CCL2-CCR2 axis represents a central pro-metastatic mechanism in lung cancer, integrating NF-κB, autophagy, and microbiome-derived signals, and highlights promising therapeutic opportunities through TLR pathway inhibition or FFAR2 restoration.

### 2.6 PI3K/Akt/mTOR pathway CCL2-CCR2 axis

**Figure 2:**
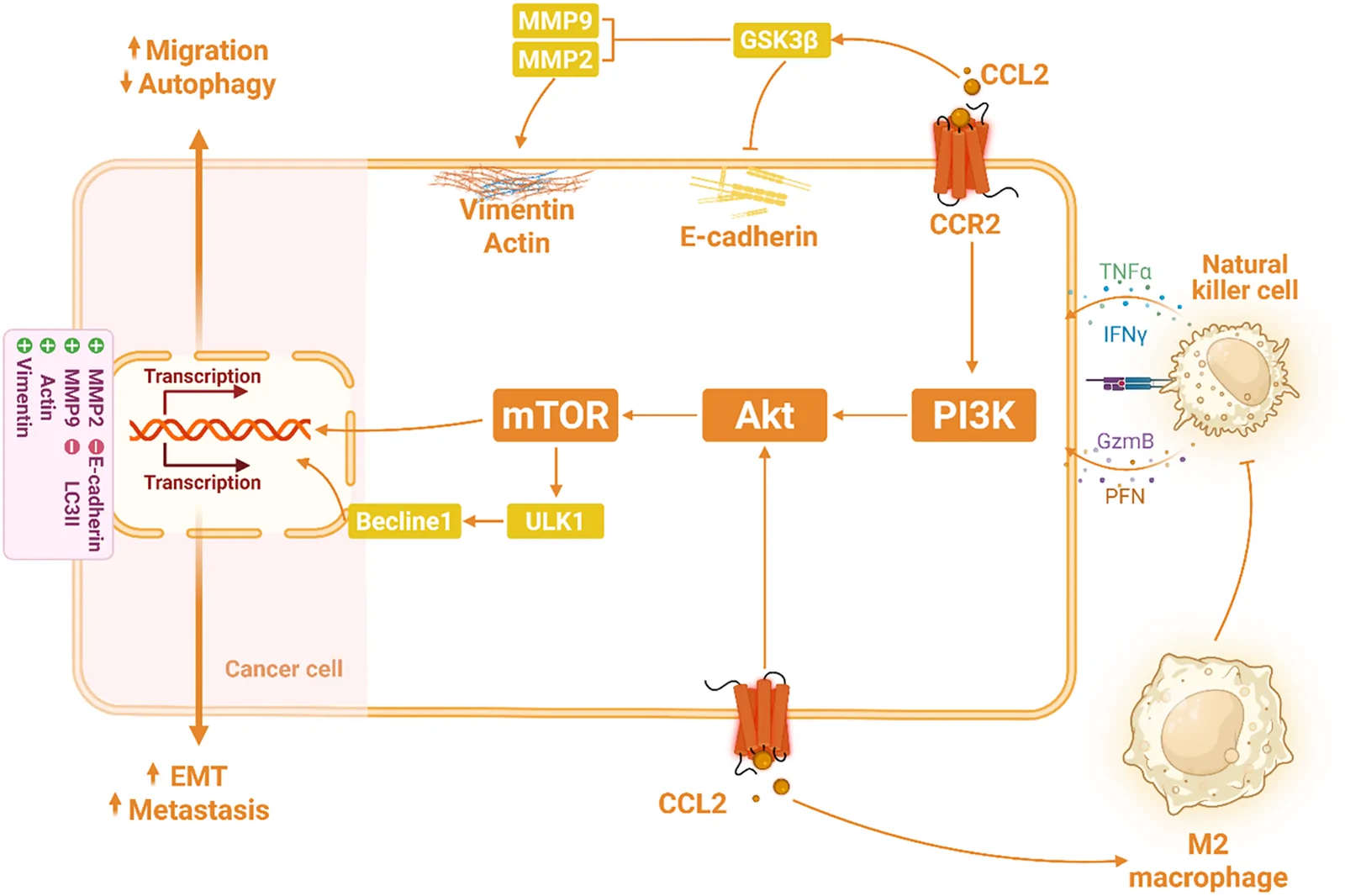
Mechanistic interplay between the CCL2–CCR2 axis and PI3K/Akt/mTOR signaling in NSCLC progression. Schematic representation illustrating how CCL2 binding to its receptor CCR2 activates the PI3K/Akt/mTOR cascade, driving EMT, cytoskeletal remodeling, and metastatic behavior in NSCLC. Activation of this pathway upregulates mesenchymal markers (vimentin, MMP-2, MMP-9) while suppressing the epithelial marker E-cadherin, thereby enhancing migratory capacity. Concurrently, PI3K/Akt/mTOR signaling disrupts autophagic flux through inhibition of key regulators such as ULK1 and Beclin1, contributing to autophagy impairment. The pathway also involves GSK3β modulation and transcriptional reprogramming that reinforce tumor progression. In the TME, CCL2 promotes recruitment and polarization of M2 macrophages and suppresses natural killer (NK) cell cytotoxicity, reducing antitumor immune responses. Collectively, these interconnected processes establish a feed-forward loop that amplifies EMT, metastasis, immune evasion, and therapeutic resistance, highlighting the CCL2–CCR2/PI3K/Akt/mTOR axis as a critical driver of lung cancer progression.

The PI3K/Akt/mTOR pathway critically modulates chemokine signaling, including the production and downstream effects of CCL2, thereby influencing inflammatory responses, immune cell recruitment, and tumor progression in lung cancer (45). In NSCLC, the CCL2-CCR2 axis directly engages and hyperactivates the PI3K/Akt/mTOR pathway to drive metastasis. Upon CCL2 binding to CCR2, PI3K is phosphorylated, leading to Akt activation and subsequent mTOR signaling. This cascade promotes EMT by upregulating mesenchymal markers, vimentin, MMP-2, MMP-9, and downregulating the epithelial marker E-cadherin in NSCLC cells overexpressing CCL2 (**Figure 2**) (33).

The CCL2-CCR2 axis disrupts autophagy in a manner that merits detailed examination. CCL2 initiates the autophagic process but impairs autophagic flux, as shown by p62 accumulation, reduced expression of Atg5, Atg7, ULK1, Beclin1, and Atg16L1, and confirmed LC3BII turnover assays. This blockade prevents the completion of autophagy, leading to the accumulation of damaged organelles and proteins that further fuel tumor progression (46). This autophagic impairment is closely linked to mitochondrial dynamics and mitophagy in lung cancer. Hyperactivation of the PI3K/Akt/mTOR pathway downstream of CCL2-CCR2 signaling suppresses mitophagy primarily through mTORC1-mediated inhibition of ULK1 and interference with PINK1/Parkin-dependent pathways. Consequently, dysfunctional mitochondria accumulate, elevating ROS levels and promoting metabolic reprogramming that enhances tumor cell survival, EMT, and resistance to therapies such as TKIs. Dysregulated mitochondrial fission can further intersect with CCL2-driven inflammatory loops, amplifying chemokine expression and TAM recruitment. This mitochondrial dimension highlights the expanding relevance of the CCL2-CCR2 axis in lung cancer pathogenesis and therapeutic resistance (47, 48).

The resulting upregulation of phosphorylated PI3K, Akt, P70S6K, ERK, GSK3β, and mTOR, alongside blocked autophagic flux, synergistically enhances EMT activation and metastatic potential, as illustrated in mechanistic models linking these events in NSCLC cells (48). Bioinformatics analyses of datasets such as GSE6013 further support this interaction, revealing enrichment of autophagy- and metastasis-related genes in pathways including PI3K-Akt and autophagy, with CCL2 emerging as a positively correlated regulator of immune infiltration. In gefitinib-resistant models, reduced β-catenin derepresses CCL2 secretion, amplifying axis activity and macrophage recruitment, which attenuates TKI efficacy, effects tied to sustained PI3K/Akt/mTOR signaling that fosters resistance and aggressive phenotypes without canonical mutations (49).

As mentioned, the bidirectional and reinforcing interaction between the CCL2-CCR2 axis and PI3K/Akt/mTOR pathway constitutes a key driver of EMT, autophagic impairment, metastasis, and therapeutic resistance in lung cancer, positioning combined targeting of this axis as a promising strategy to disrupt tumor progression.

### 2.7 The role of STAT3 in CCL2/CCR2 regulation

STAT3 functions as a central transcription factor that regulates chemokine networks within the tumor immune microenvironment, directly controlling CCL2 expression to shape inflammatory and immunosuppressive responses in lung cancer (50).

In NSCLC, IL-7/IL-7R signaling activates the JAK1/STAT3 pathway in tumor cells, transcriptionally upregulating CCL2 production (51). *In vitro* experiments confirmed that IL-7 stimulation markedly elevates CCL2 mRNA and protein levels, an effect completely abolished by JAK1/STAT3 pathway inhibitors. Immunohistochemical examination of specimens from 119 NSCLC patients further demonstrated strong positive correlations between high IL-7/IL-7R expression, elevated CCL2, and increased CD11b+ MDSC infiltration. These features aligned with advanced clinical stage, poorer tumor differentiation, and shorter overall survival, with multivariate analysis establishing CCL2, IL-7R, differentiation degree, and tumor stage as the strongest independent predictors of patient outcome. Consistent with earlier findings that high IL-7/IL-7R portends poor prognosis, this axis integrates with the known tumor-promoting and immunosuppressive roles of JAK1/STAT3, which drive MDSC amplification at tumor sites in NSCLC and related malignancies (52). Building on these observations, *in vivo* studies using Lewis lung carcinoma (LLC) mouse models showed that IL-7 administration significantly enhanced MDSC recruitment to tumor tissues through CCL2 secretion. This recruitment was effectively blocked by IL-7R antagonism or CCR2 inhibitors such as RS102895, resulting in reduced tumor growth rates, lower Ki-67 proliferation indices, and decreased intratumoral MDSC numbers. Complementary MDSC depletion with anti-Gr-1 antibodies similarly reversed IL-7-driven tumor progression, underscoring the CCL2/CCR2-mediated mechanism. Notably, the CCL2/CCR2 axis also cooperates with IL-6 to synergistically phosphorylate STAT3, establishing a positive feedback loop in which activated STAT3 further induces both IL-6 and CCL2 production. This amplification potentiates EMT primarily by upregulating Twist transcription, thereby enhancing tumor cell invasion in NSCLC (53).

Ultimately, STAT3 serves as both an upstream regulator and downstream effector within the CCL2/CCR2 network, orchestrating MDSC recruitment, immunosuppression, and metastatic progression in lung cancer while highlighting the pathway’s potential as a multifaceted therapeutic target.

### 2.8 NcRNAs interaction with CCL2/CCR2 axis

Non-coding RNAs, including microRNAs and circular RNAs, emerge as key orchestrators of chemokine regulation within the lung cancer TME, fine-tuning CCL2 expression and CCR2-dependent signaling to drive oncogenic processes (54, 55). In LUAD, circHSPB6 is markedly overexpressed and operates through a ceRNA network with let-7a-2-3p and CCL2. Acting as a molecular sponge, circHSPB6 sequesters let-7a-2-3p, thereby relieving its repressive effect on CCL2 mRNA and elevating chemokine production. This circHSPB6/let-7a-2-3p/CCL2 feedback loop sustains high CCL2 levels, which in turn promote LUAD cell proliferation, migration, and invasion while suppressing apoptosis *in vitro* and accelerating xenograft tumor growth *in vivo*. Critically, CCL2 secreted under circHSPB6 control also stimulates M2 polarization and infiltration of TAMs; these reprogrammed TAMs further amplify EMT, creating a self-reinforcing pro-metastatic niche (56). Parallel stromal reprogramming is mediated by miR-196a within CAFs. Highly expressed in the tumor stroma and linked to poor prognosis in lung adenocarcinoma, miR-196a directly targets the 3′-UTR of ANXA1, an anti-inflammatory protein that normally represses CCL2 transcription via inhibition of pro-inflammatory pathways. miR-196a in CAFs targets ANXA1, unleashing CCL2 secretion and conferring both myofibroblastic and inflammatory traits (57).

A key mechanism involves hypoxia-induced upregulation of miR-210-3p, a prominent hypoxamiR, which directly targets the 3’-UTR of CCL2 mRNA, thereby suppressing CCL2 expression. This downregulation impairs monocyte chemoattraction and infiltration into the tumor, as CCL2 acts as a potent attractant via the CCR2 axis (58). Experimental evidence from *in vitro* hypoxia models (1% O_2_ versus normoxia at 21% O_2_, with physoxia around 5-6% in tissues) demonstrates that HIF-1α stabilization elevates miR-210-3p levels, leading to reduced CCL2 and diminished monocyte migration. Conversely, HIF-1α silencing or miR-210-3p inhibition restores CCL2 expression and monocyte recruitment. Luciferase assays confirm direct binding, abolished by seed-region mutations, while rescue experiments with miR-210-3p mimics in HIF-1α-silenced cells or inhibitors in overexpressed cells further validate this axis. Thus, the HIF-1α/miR-210-3p/CCL2 axis represents a critical immunosuppressive pathway in hypoxic LUAD, highlighting miR-210-3p inhibition as a promising immunomodulatory strategy to enhance monocyte infiltration, reprogram TAMs toward tumoricidal phenotypes, and improve therapeutic outcomes in lung cancer and potentially other solid tumors (59).

Although numerous miRNAs interact with the CCL2/CCR2 axis in other cancer types, several have also been linked to CCL2 regulation or related chemokine-driven processes specifically in lung cancer. For instance, miR-1 is consistently downregulated in SCLC tissues and serum; it suppresses tumor growth and metastasis partly by targeting CXCR4, thereby modulating chemokine-responsive pathways that intersect with CCL2-CCR2 signaling (60). Similarly, miR-206, miR-146a-5p, and miR-196b-5p influence inflammatory and STAT3-linked circuits in NSCLC that converge on CCL2-mediated macrophage recruitment and immunosuppression, highlighting their potential contribution to the axis in lung cancer contexts (61–63).

**Figure 3:**
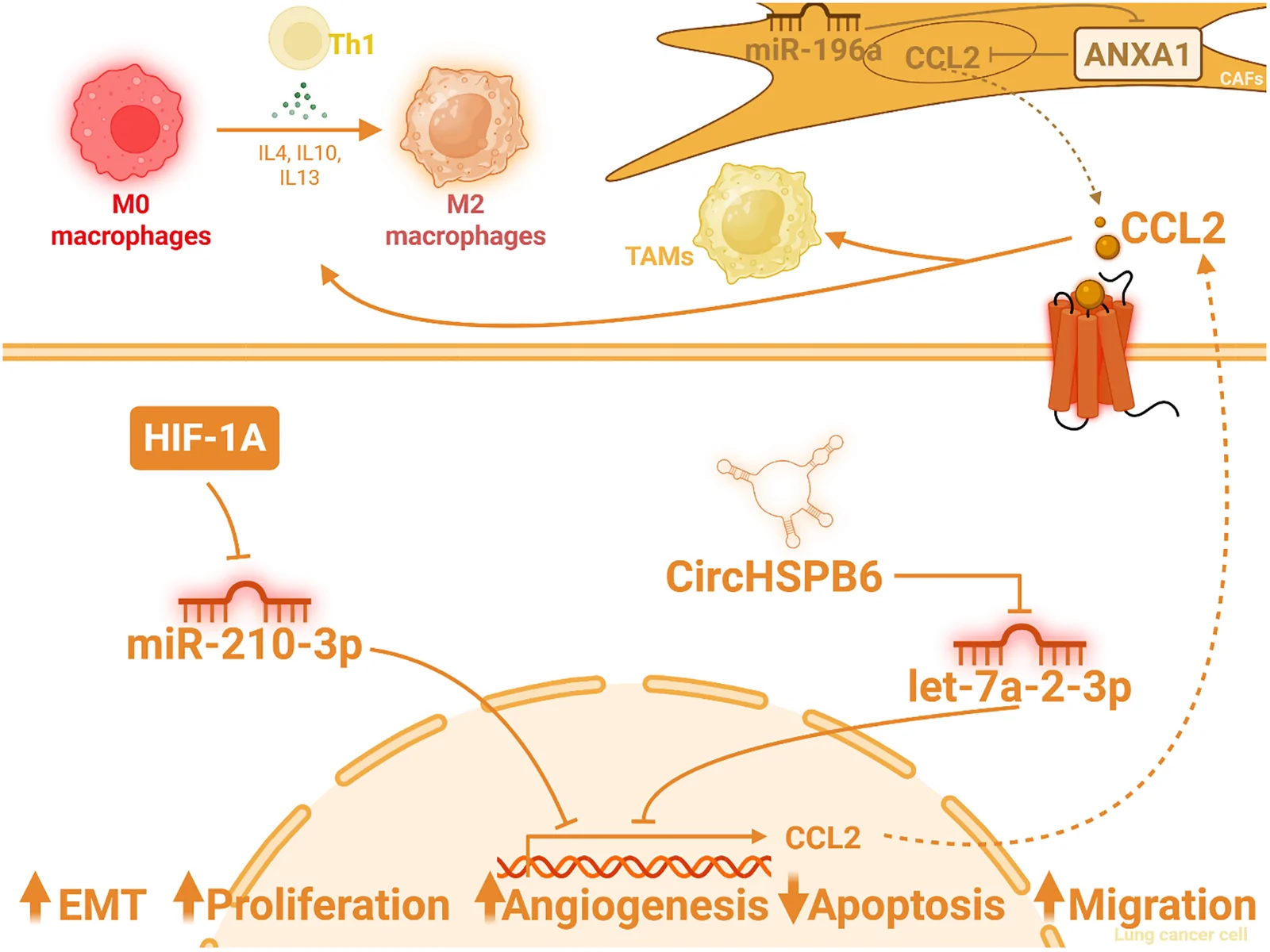
NcRNA-mediated regulation of the CCL2/CCR2 axis in the lung cancer microenvironment. This schematic representation illustrates how non-coding RNAs coordinately modulate CCL2-driven signaling across tumor, stromal, and immune compartments in lung adenocarcinoma. circHSPB6 functions as a competing endogenous RNA by sequestering let-7a-2-3p, thereby relieving repression of CCL2 and enhancing its expression. Elevated CCL2 promotes tumor cell proliferation, migration, angiogenesis, and EMT, while inhibiting apoptosis. Tumor-derived CCL2 further recruits and polarizes macrophages toward an M2 phenotype, generating TAMs that reinforce a pro-metastatic niche. In parallel, CAFs contribute to CCL2 upregulation via miR-196a-mediated suppression of ANXA1, amplifying inflammatory and invasive signals within the tumor stroma. Under hypoxic conditions, HIF-1α induces miR-210-3p, which directly targets CCL2 mRNA and reduces its expression, thereby limiting monocyte recruitment. Together, these interconnected ncRNA networks dynamically regulate the CCL2/CCR2 axis, shaping tumor progression, immune cell infiltration, and metastatic potential.

From a clinical perspective, these ncRNAs hold significant translational value as potential biomarkers. For example, elevated circHSPB6 and miR-196a expression have been associated with advanced disease stages and poor prognosis in lung adenocarcinoma, while circulating miR-1 levels in SCLC correlate with tumor burden and metastatic potential. Such ncRNA signatures could complement existing markers for early detection, risk stratification, and monitoring therapeutic responses targeting the CCL2-CCR2 axis, thereby advancing precision oncology approaches in lung cancer. In conclusion, ncRNA-directed control of the CCL2/CCR2 axis integrates epithelial, stromal, and immune compartments to fuel lung cancer progression, positioning circHSPB6, let-7a-2-3p, and miR-196a as promising molecular targets for disrupting tumor-stroma crosstalk and improving therapeutic outcomes (**Figure 3**).

### 2.9 Conceptual convergence of upstream regulators on the CCL2–CCR2 signaling network

Although the upstream regulators described in this section originate from diverse biological processes, including metabolic reprogramming (PRPS2), transcriptional regulation (CtBP1 and STAT3), growth factor signaling (PDGF-BB), innate immune activation (TLRs), PI3K/Akt/mTOR signaling, stromal remodeling by cancer-associated fibroblasts, and ncRNA-mediated post-transcriptional regulation, they ultimately converge on a remarkably conserved biological program centered on the CCL2–CCR2 axis (17, 18). Rather than functioning as isolated signaling events, these pathways collectively enhance CCL2 production or potentiate CCR2 signaling, thereby establishing a common molecular framework that governs communication between tumor cells and the surrounding microenvironment. This convergence transforms the CCL2–CCR2 axis into a central signaling hub that integrates oncogenic, inflammatory, stromal, and metabolic cues to coordinate disease progression (9, 32, 33).

Importantly, despite their mechanistic diversity, these upstream signals activate a limited number of shared downstream biological processes. Increased CCL2 expression promotes CCR2-dependent recruitment of circulating monocytes, which subsequently differentiate into tumor-associated macrophages and monocytic myeloid-derived suppressor cells while simultaneously enhancing interactions with cancer-associated fibroblasts and other stromal components. The accumulation of these immunosuppressive populations establishes a feed-forward inflammatory network characterized by sustained secretion of cytokines, chemokines, matrix-remodeling enzymes, and angiogenic mediators (17, 24, 25). Consequently, immune surveillance is progressively attenuated through suppression of cytotoxic T-cell and natural killer cell activity, whereas extracellular matrix degradation, epithelial-mesenchymal transition, angiogenesis, and metastatic dissemination are collectively reinforced. These interconnected mechanisms further illustrate that the CCL2–CCR2 axis operates as a dynamic amplifier rather than a simple chemotactic pathway. Recruited myeloid cells, activated fibroblasts, and tumor cells continuously reinforce one another through reciprocal paracrine signaling, maintaining persistent activation of downstream pathways such as NF-κB, PI3K/Akt/mTOR, and STAT3 (43, 52). These self-sustaining feedback circuits stabilize the immunosuppressive tumor microenvironment, facilitate therapeutic resistance, and support metastatic colonization even when the initiating upstream stimulus differs substantially. Thus, the biological significance of the CCL2–CCR2 axis lies less in any individual upstream regulator than in its ability to integrate multiple oncogenic and inflammatory signals into a unified tumor-promoting network (49).

Nevertheless, the biological effects of CCL2–CCR2 signaling remain context-dependent, as demonstrated by counter-regulatory mechanisms such as Nrf2-mediated TAM repolarization and restoration of antitumor immune responses in specific lung cancer settings (23). Overall, the evidence indicates that although multiple upstream regulators independently influence this pathway, their predominant consequence is convergence on a shared CCL2–CCR2-centered network that promotes myeloid recruitment, immune evasion, EMT, angiogenesis, metastasis, and therapy resistance. Therefore, targeting this axis as an integrative signaling hub, rather than focusing on individual upstream molecules, may represent a more effective therapeutic strategy for disrupting lung cancer progression.

## 3 Metastasis

### 3.1 Lung cancer metastasis

The CCL2-CCR2 axis serves as a central driver of lung cancer metastasis, particularly in NSCLC subtypes such as LUAD and squamous cell carcinoma (LUSC). By promoting the recruitment and polarization of TAMs, this chemokine-receptor interaction reshapes the TME to enhance cancer cell invasion, EMT, extracellular matrix remodeling, and distant spread (74).

Overexpression of the long non-coding RNA MALAT1 in LUAD cells triggers a non-cell-autonomous mechanism whereby elevated CCL2 secretion recruits M2-polarized macrophages. This leads to stromal remodeling, increased cellular mobility, and progression to poorly differentiated, metastatic disease. MALAT1 acts through interference with PRC2 function and global chromatin accessibility changes, de-repressing CCL2 expression. Consequently, Ccl2 blockade in preclinical models reverses macrophage recruitment, reduces tumor burden and grade, and limits metastasis, underscoring the axis as a key effector of MALAT1-driven progression (75). In parallel, ERα in NSCLC cells up-regulates CCL2 transcription, fostering macrophage infiltration, M2 polarization, and MMP9 secretion that collectively promote cancer cell invasion. Infiltrating macrophages establish a positive feedback loop by secreting CXCL12, which activates CXCR4 on tumor cells, elevating p-ERK1/2 and p-Akt signaling to further increase ERα levels. This ERα/CCL2/CCR2/MMP9 and CXCL12/CXCR4 circuit operates prominently in early-stage disease across both sexes and is reversed by CCL2 knockdown or CCR2 antagonism (10).

The oncogenic splice variant KLF6-SV1 similarly engages the axis by transcriptionally up-regulating TWIST1 and CCL2, thereby accelerating EMT, elevated N-cadherin and MMP-9, reduced E-cadherin, and M2 macrophage polarization (increased CD163, decreased CD68) via M-CSF modulation. In co-culture systems, M2 macrophages amplify KLF6-SV1-driven invasion, while TWIST1 silencing attenuates CCL2 expression and metastatic potential, revealing a TWIST1-CCL2-TAM circuit that links KLF6-SV1 to lung cancer dissemination (76). Conversely, the transcription factor TSHZ3 functions as a tumor suppressor in LUAD by leveraging the CCL2-CCR2 axis for anti-metastatic effects. Its overexpression elevates CCL2 in cancer cells and CCR2 in macrophages, recruiting CD86+ M1-polarized macrophages that induce tumor cell apoptosis in co-culture without affecting viability in monoculture. This immune-mediated restraint of EMT, migration, and distant metastasis correlates with favorable prognosis and enrichment of inflammatory and immune-response pathways (77).

In summary, the CCL2-CCR2 axis integrates oncogenic, MALAT1, ERα, KLF6-SV1, and suppressive, TSHZ3, signals to dictate macrophage polarization and metastatic fate in lung cancer. Its context-dependent duality highlights promising therapeutic avenues, including CCL2 blockade or upstream targeting, to disrupt pro-metastatic crosstalk while preserving anti-tumor immunity.

### 3.2 Breast to lung metastasis

In breast cancer, particularly its aggressive triple-negative subtype, the role of CCL2/CCR2 Axis in Breast cancer to lung metastasis stands out as a central mediator that hijacks monocytic cells to establish a supportive pre-metastatic niche and drive pulmonary colonization. Tumor- and stroma-derived CCL2 binds CCR2 on inflammatory monocytes, accelerating their recruitment to the lungs while simultaneously reprogramming distant sites for immune evasion and tumor cell extravasation (78).

This axis operates through distinct, organ-tuned mechanisms. In MDA-MB-231-derived lung-metastatic models, CCL2 overexpression markedly speeds metastatic seeding within the first week by recruiting mature F4/80+ macrophages that enhance invasion and early micrometastatic cluster formation; thereafter, growth rates parallel those of controls, indicating CCL2’s primary benefit at initial lodging rather than sustained expansion. Primary cells from CCL2- and CCR2-knockout mice confirmed that tumor-derived CCL2 directs macrophage migration and, by extension, creates the permissive lung microenvironment in a strictly CCR2-dependent fashion. Neutralizing CCL2 antibodies correspondingly suppress lung colony formation (79). Parallel analyses of tumor-draining lymph nodes (TDLNs) in triple-negative models further reveal that resident fibroblasts (particularly medullary reticular cells and TRCs) up-regulate CCL2 and CCL7, attracting CCR2-expressing monocytes that differentiate into programmed death-ligand 1 (PD-L1+)/iNOS+ immunosuppressive cells functionally akin to monocytic myeloid-derived suppressor cells; these monocytes blunt T-cell proliferation, thereby shielding disseminating tumor cells en route to the lungs (80). Chemotherapy introduces additional complexity by paradoxically exacerbating lung metastasis through host-mediated effects involving the CCL2/CCR2 pathway. Agents like gemcitabine, paclitaxel, and doxorubicin trigger reactive myelopoiesis in the bone marrow, boosting monocyte production via elevated mitochondrial ROS in hematopoietic progenitors; this process is mitigated by ROS scavengers such as mitoTEMPO. The resulting CCR2-dependent accumulation of bone marrow-derived interstitial macrophages/MAMs in the lungs drives pro-metastatic remodeling, independent of primary tumor presence. Notably, chemotherapy upregulates coagulation factor X (FX, encoded by F10) in lung macrophages, fostering a hypercoagulable, fibrotic microenvironment that promotes metastatic outgrowth through extracellular matrix remodeling and potentially impaired antitumor immunity. Inhibiting FX with rivaroxaban or macrophage-specific F10 knockdown substantially attenuates this chemotherapy-induced metastatic promotion, highlighting FXa as a combinatorial target alongside anticoagulation (81).

Small extracellular vesicles carrying caveolin-1 (CAV1) from primary breast tumors reach lung epithelium, up-regulating integrin α6β4 signaling and, via the TLR4/NF-κB route, inducing IL-6 and CCL2 release; the latter recruits and polarizes tumor-associated neutrophils toward the pro-metastatic N2 phenotype through NLRP3 inflammasome activation, while simultaneously promoting epithelial–mesenchymal transition and angiogenesis through VEGF-A and MMP9 (82). Further nuance arises from therapeutic targeting of the axis itself. While CCL2 neutralization sequesters monocytes in the bone marrow and curbs metastasis, abrupt interruption of blockade triggers a rebound: monocyte release, enhanced primary tumor mobilization, pulmonary angiogenesis, and accelerated metastatic proliferation in an IL-6- and VEGF-A-dependent fashion, ultimately hastening death in syngeneic models. This underscores the TME’s influence on therapy efficacy and cautions against anti-CCL2 monotherapy in metastatic settings, favoring sustained or combined inhibition (e.g., with IL-6 blockade) to prevent overshoot (83, 84).

As evidenced in **Table 2**, the CCL2/CCR2 axis integrates monocyte recruitment, macrophage polarization, chemotherapy-triggered host responses, and coagulation dysregulation to propel breast cancer lung metastasis. These multifaceted roles reinforce its status as a high-priority therapeutic target, yet emphasize the need for careful, context-aware strategies to maximize benefits while averting counterproductive rebound effects in clinical translation.

**Table 2:** Overview of CCL2-CCR2 signaling in breast cancer lung metastasis: key studies and mechanisms.

Type of metastasis	Role of CCL2 (pro or anti-metastatic)	CCL2-CCR2 activity	Type of study	Cancer and cell lines	Pathway	Highlights	Ref
Breast to lung	Pro-metastatic	↑ CCL2-CCR2	*In vitro* and *in vivo*	Human breast cancer cells (MDA-MB-435, MDA-MB-231, T47D, and MCF7); lung metastatic foci (E0771-LG)	CCL2-CCR2/CCL3-CCR1/metastasis-associated macrophages (MAMs)	“CCL2-triggered chemokine cascade in macrophages promotes metastatic seeding of breast cancer cells.”	(85)
Breast to lung	Pro-metastatic	↑ CCL2	*In vivo*	Murine breast cancer cells (4T1); subline 4T1-L10	Stromal CCL2/macrophage recruitment/angiogenesis/tumor-derived CCL2/seeding/survival in lung	“Stromal (non-tumor) CCL2 is main driver of spontaneous lung metastasis via macrophage influx & angiogenesis.”	(86)
Breast to lung	Pro-metastatic	↑ CCL2	*In vitro* and *in vivo*	Murine breast cancer cells (4T1); human MDA-MB-231; lung fibroblasts (mouse & HFL1 human)	LC3+ EVs/HSP60/TLR2-MyD88-NF-κB/fibroblast CCL2/monocyte recruitment/PMN	“Tumor-released LC3+ EVs carry HSP60, stimulate lung fibroblasts to secrete CCL2, recruit monocytes, suppress T-cell function, increase vascular leakage, and promote lung premetastatic niche (PMN).”	(87)
Breast to lung	Pro-metastatic	↑ CCL2	*In vitro* and *in vivo*	Murine breast cancer cells (4T1, AT-3); lung-derived MSCs (LMSCs) from tumor-bearing mice	Sca-1high LMSCs/CCL2, CCL7, CXCL1/myeloid cell recruitment + direct tumor cell attraction	“Sca-1high lung MSCs express higher CCL2/CCL7/CXCL1, recruit macrophages (CCR5-dependent) and tumor cells (CXCL1), promoting lung metastatic colonization.”	(88)
Breast to lung	Pro-metastatic	↑ CCL2-CCR2	*In vitro* and *in vivo*	Mouse breast cancer models (4T1, 67NR, PyMT)	CCL2-CCR2/neutrophils (TEN), macrophages, CD4+ & CD8+ T cells	“Intranasal CCL2 increased lung seeding/outgrowth of 67NR cells, recruited CD4+ T cells & central memory CD8+ T cells, balanced CD206+/MHCII+ macrophages.”	(89)
Breast to lung	Pro-metastatic	↑ CCL2-CCR2	*In vivo*	4T1 (mouse mammary carcinoma); MMTV-PyMT spontaneous model; human breast cancer lung metastases	CCL2- CCR2/MHCIIhi neutrophil recruitment	“Chronic pulmonary bacterial infection upregulates lung CCL2, which recruits tumor-promoting MHCIIhi neutrophils to form pre-metastatic niche and enhance lung metastasis.”	(90)

### 3.3 CRC to lung metastasis

In CRC progressing to pulmonary sites, the CCL2/CCR2 axis emerges as a central driver of metastatic colonization. Tumor- and stroma-derived CCL2 recruits CCR2-expressing inflammatory monocytes, which differentiate into TAMs that sustain an inflammatory microenvironment conducive to extravasation and growth. The role of CCL2/CCR2 Axis in CRC cancer to lung metastasis is further amplified by macrophage-intrinsic regulators such as APOC1, which enhance chemokine output and tumor cell adaptation (91).

Molecularly, CRC cells secrete CCL2 that engages endothelial CCR2, triggering vascular permeability and enabling tumor cell transmigration. Concurrently, CCR2-deficient monocytes fail to respond to tumor-derived supernatants *in vitro*, underscoring direct dependence on this pathway for initial recruitment (92). In MC38 and LLC1.1 models of CRC lung metastasis, Ccl2 knockdown markedly reduces macrophage (F4/80+) and myeloid cell infiltration, correlating with attenuated metastatic burden even in Ccr1-deficient hosts. Adoptive transfer experiments reveal nuance: Ccr2-deficient monocytes, once circulating, are still efficiently recruited to early metastatic lungs via alternative Ccr1-mediated mechanisms, rescuing metastasis in Ccr1-null recipients; conversely, Ccr1-deficient monocytes do not restore progression in Ccr2-null animals. This indicates sequential, non-redundant action wherein CCL2/CCR2 initiates monocyte egress and CCR1 sustains site-specific retention and macrophage differentiation (93). Macrophages expressing APOC1 in CRC pulmonary metastases (CCPM) intensify this cascade by activating STAT3, which transcriptionally upregulates CCL2 and CCL5 secretion. These chemokines promote CRC cell EMT, invasion, and angiogenesis while recruiting additional TAMs. Single-cell data confirm APOC1 enrichment in lung-resident macrophages, with knockdown via macrophage-targeted AAV vectors or clodronate depletion abolishing metastatic enhancement *in vivo*. Notably, CCL2/CCR5 and CCL5/CCR1 axes further modulate Treg recruitment and M2 polarization, creating immunosuppression that favors colonization. Although Ccr2 deficiency alone reduces circulating monocytes and TAMs, combined Ccl2 knockdown in Ccr1-null settings virtually eliminates lung metastases, highlighting convergent chemokine signaling (94).

A promising alternative involves CCL2 decoy strategies, such as the dnCCL2-HSA chimera, which exhibits enhanced glycosaminoglycan binding and CCR2 antagonism. Unlike systemic CCR2 inhibitors that deplete circulating monocytes via bone marrow effects, this chimera localizes to tumor-activated lung endothelium, upregulating syndecan-4 (SDC4) in response to tumor signals and TNF-α, without altering peripheral leukocyte counts. It inhibits vascular permeability, reduces CD11b+ cell association with tumor cells, and blocks monocyte-dependent transendothelial migration *in vitro*, culminating in attenuated seeding and metastasis of CRC-derived lines *in vivo* (95). These interconnected mechanisms position the CCL2/CCR2 axis as a multifaceted therapeutic vulnerability in CRC lung metastasis, where targeted disruption of chemokine secretion, receptor engagement, or downstream STAT3 signaling could impair monocyte recruitment, vascular remodeling, and tumor cell adaptation to achieve durable control.

### 3.4 Melanoma to lung metastasis

The role of CCL2/CCR2 Axis in Melanoma cancer to lung metastasis emerges as a critical driver of pulmonary colonization by melanoma cells, particularly through the creation of a permissive pre-metastatic niche that supports circulating tumor cell engraftment and outgrowth (96).

In syngeneic models employing B16F10 melanoma cells, tumor-derived secretory osteopontin (sSPP1) acts upstream of this axis by inducing CCL2 production, which in turn engages host CCR2 receptors to promote lung homing and metastatic seeding (97). This isoform-specific signaling operates independently of primary-site dissemination yet is essential for late-stage colonization, as demonstrated by enhanced metastatic burden following sSPP1 transcript overexpression in otherwise osteopontin-deficient B16F10 cells. The pathway converges on endothelial activation and immune modulation, reconciling earlier observations of osteopontin–CCL2 crosstalk while highlighting its dispensability in certain host contexts where local tumor-derived levels predominate (98). These effects are amplified by circulating fibrocytes, which precondition the pulmonary stroma prior to tumor arrival. Adoptive transfer of wild-type fibrocytes into CCR5-deficient recipients prior to B16F10 injection markedly increases lung metastases by triggering CCL2 secretion, thereby recruiting Ly-6C+ monocytes that differentiate into immunosuppressive macrophages. This process depends on MMP9 activity and establishes a positive feedback loop wherein CCL2 further mobilizes fibrocytes and monocytes, while CCR2 signaling on endothelial cells facilitates tumor cell extravasation. Notably, fibrocyte numbers remain low, yet their impact is profound, distinguishing them from other stromal precursors and explaining prometastatic effects observed after surgical wound healing, when fibrocyte mobilization peaks (99). Complementing these mechanisms, tumor-derived microparticles (T-MPs) released from melanoma cells are internalized by resident lung macrophages, prompting CCL2 release that recruits additional monocytes and polarizes them toward both M1 and M2 phenotypes. The resulting IL-6 surge sustains tumor-repopulating cells, highly tumorigenic subpopulations capable of forming colonies in soft 3D matrices, while concurrent inflammatory signals increase capillary permeability, leading to fibrin(ogen) deposition. Deposited fibrin delivers mechanical cues via integrin-mediated mechanotransduction, further enhancing survival and outgrowth of stem-like melanoma cells (100).

These interconnected pathways illustrate how the CCL2/CCR2 axis integrates cytokine, cellular, and biophysical signals to orchestrate melanoma lung metastasis, providing a compelling rationale for isoform-selective osteopontin inhibition, dual CCR2/CCR5 blockade, or MMP9-targeted strategies to disrupt niche formation and improve therapeutic outcomes.

### 3.5 Osteosarcoma to lung metastasis

Osteosarcoma (OS) frequently metastasizes to the lungs, where the CCL2-CCR2 axis serves as a central driver of disease progression. Tumor-derived CCL2 exploits this chemokine-receptor interaction to recruit and polarize immunosuppressive cells, thereby establishing a permissive pre-metastatic niche that accelerates metastatic colonization and contributes to the limited efficacy of current therapies (101).

At the molecular level, metastatic OS cells, exemplified by murine LM8 and human 143B lines, secrete high levels of CCL2, a substantial portion of which is encapsulated within extracellular vesicles (EVs) (102). These CCL2-positive EVs preferentially traffic to the lungs and are internalized by CCR2-expressing macrophages, triggering their differentiation into pro-tumorigenic M2-like phenotypes. This reprogramming is mediated through CCL2-CCR2 signaling, which activates pathways such as JAK/STAT and STAT6, while synergizing with other metastasis-associated factors including M-CSF and CXCR2 ligands, CXCL1, CXCL2, and CXCL8, that further amplify macrophage recruitment and polarization. Consequently, M2-like macrophages accumulate in lung tissue, fostering an immunosuppressive microenvironment that supports OS cell seeding and outgrowth. Clinical correlation is evident in patient specimens, where elevated CCL2 expression in primary tumors and lung metastases strongly associates with increased M2 macrophage infiltration. Pharmacologic interruption of the axis via CCL2-neutralizing antibodies in orthotopic models markedly reduces EV accumulation and lung metastases without affecting primary tumor growth, confirming the axis’s selective role in metastatic dissemination rather than primary tumorigenesis (103). In contrast to its pathogenic function, the CCL2-CCR2 axis can be therapeutically redirected. Engineered bacterial vectors such as VNP-C-C, which co-express CCL2 and CXCL9, harness tumor tropism to deliver these chemokines directly into the metastatic site. Beyond direct oncolytic and anti-angiogenic effects, VNP-C-C induces immunogenic cell death in OS cells, activates the cGAS/STING pathway, and secretes proinflammatory cytokines that promote M1 macrophage polarization, dendritic cell maturation, and T-cell infiltration—even in T-cell-deficient models. This remodeling reverses local immunosuppression, reduces CD4⁻CD8⁻ double-negative T cells, upregulates PD-1 on effector T cells, and elicits systemic immunity through antigen presentation in the spleen, ultimately decreasing metastatic burden and extending survival (104).

Taken together, the CCL2-CCR2 axis orchestrates osteosarcoma lung metastasis through tumor-driven M2 macrophage accumulation while simultaneously offering a versatile target for engineered immunotherapies that reprogram the same pathway toward antitumor immunity, paving the way for precision combination strategies.

### 3.6 Renal cell carcinoma to lung metastasis

Renal cell carcinoma (RCC) leads to most deaths via lung metastasis mediated by tumor-derived EVs carrying C3 (105). Ingested by tissue-resident lung macrophages, these EVs activate TAMs, priming pre-metastatic niches through angiogenesis, matrix remodeling, vascular leakiness, and immunosuppression while recruiting monocytes and myeloid cells. Macrophages, the most abundant stromal cells in the TME, drive protumorigenic functions including invasion and antitumor-immune suppression; selective targeting strategies such as anti-CSF1R antibodies or Irg1 deficiency enhance T-cell responses. The CCL2-CCR2 axis plays a pivotal role and interaction in RCC to lung metastasis, as EV C3-activated TAMs secrete CCL2 to recruit inflammatory monocytes via CCR2, promoting tumor cell extravasation and TAM polarization. It interacts with the CXCL1/CXCR2 axis for PMN-MDSC recruitment, yet single-axis blockade is inferior to C3 knockout, underscoring EV C3 as the upstream initiator of both chemokine networks. Then, targeting the CCL2-CCR2 axis triggered by tumor EV C3 holds potential to inhibit immunosuppressive myeloid-driven metastasis in RCC (106).

## 4 Therapeutic approaches targeting CCL2-CCR2 chemokine axis

### 4.1 Immunotherapy

#### 4.1.1 CCL2-CCR2 axis in CAR-T cell therapy

The CCL2-CCR2 axis has emerged as a key regulator that augments CAR-T cell therapy by directing engineered T cells along chemokine gradients to improve tumor infiltration and persistence (107). In NSCLC, B7-H3-targeted CAR-T cells effectively eliminate tumor cell lines and patient-derived organoids *in vitro* while suppressing growth in metastatic and orthotopic xenotransplant models, with comparable efficacy regardless of CD28 or 4-1BB costimulation domains. B7-H3 represents a compelling target alongside mesothelin, HER2, EGFR, and GD2, given its elevated expression across tumors and minimal presence in normal tissues, supported by clinical safety data from meningioma treatment. Intravenous delivery, however, shows limited control of brain metastases owing to the blood-brain barrier. NSCLC cells and brain metastases produce CCL2, mirroring its astrocyte-derived role in recruiting T cells during experimental autoimmune encephalomyelitis; co-expression of CCR2b in B7-H3.CAR-T cells harnesses this gradient to markedly enhance migration, intratumoral accumulation, and regression of brain-localized lesions. This effect persists even when primary lung tumors remain, remains tumor-specific with no accumulation in contralateral brain tissue, and outperforms broader chemokine strategies such as CXCR2 (whose multiple ligands raise toxicity concerns) while building on prior CCR2 or CCR4 successes in neuroblastoma, mesothelioma, and Hodgkin lymphoma. In contrast, benefits are modest in liver-dominant metastatic models where chemokine cues play a lesser role (108). Tailoring CCR2b to the CCL2-CCR2 axis offers a precise, translatable strategy to overcome barriers in CAR-T therapy and eradicate lethal NSCLC brain metastases.

#### 4.1.2 CCL2-CCR2 targeting to enhance immune checkpoint inhibitors

The CCL2-CCR2 axis serves as a central but dual regulator of macrophage recruitment and polarization within the tumor immune microenvironment, thereby exerting complex, context-dependent effects on the efficacy of ICIs across multiple malignancies. Blockade can enhance responses by reducing immunosuppression but risks impairing beneficial recruitment in some settings. In lung cancer, this chemokine-receptor interaction modulates the balance between immunosuppressive TAMs and effector T-cell activity, offering a strategic target to overcome ICI resistance and amplify therapeutic responses (109).

In NSCLC, PIM1 proto-oncogene signaling drives CCL2 expression through NF-κB-mediated transactivation in tumor cells, fostering TAM infiltration and M2-like polarization that sustains an immunosuppressive milieu. Genetic or pharmacologic ablation of PIM1 disrupts this pathway, reducing CCL2 secretion and preventing pro-tumoral macrophage reprogramming. Consequently, dual blockade of PIM1 and PD-1 synergistically suppresses tumor growth, repolarizes macrophages toward an anti-tumoral state, and markedly improves anti-PD-1 therapy outcomes by reshaping the TME (110). Parallel investigations highlight ALKBH5-mediated N6-methyladenosine (m6A) demethylation as an upstream activator of CCL2 and CXCL10 transcription in NSCLC cells. This process recruits PD-L1-expressing TAMs, promotes their M2 polarization, and upregulates PD-L1 on both tumor cells and macrophages via Janus kinase 2 (JAK2)/signal transducer and activator of transcription 3 (STAT3) signaling. Recruited TAMs further secrete IL-6, reinforcing the pathway in a feed-forward loop that maintains immunosuppression. Elevated ALKBH5 expression, however, paradoxically sensitizes tumors to anti-PD-L1 antibodies, as evidenced by enhanced overall survival in patients with PD-L1-positive TAMs receiving checkpoint blockade, underscoring the axis’s dual role in ICI responsiveness (111).

Therapeutic application of tumor-treating fields (TTFields) combined with anti-PD-1 further exploits the CCL2-CCR2 axis through induction of immunogenic cell death. This triggers IFN-γ and NF-κB activation, which upregulates CCL2/8-CCR2 and CXCL9/10-CXCR3 signaling via STAT1/IRF-1, thereby promoting robust infiltration of CD8+ and CD4+ T cells. Notably, the TTFields-plus-anti-PD-1 regimen outperforms TTFields-plus-anti-PD-L1 by more effectively remodeling the tumor immune microenvironment, highlighting context-dependent synergy between CCL2-CCR2 engagement and specific ICI modalities (112). In models of acquired resistance to PD-1/anti-cytotoxic T-lymphocyte-associated protein 4 (CTLA-4) combination therapy, IFN-γ-dependent CCL2-CCR2 signaling recruits classical lymphocyte antigen 6 complex locus C^+^ (Ly6C^+^) monocytes that accumulate as immunosuppressive populations, limiting dendritic cell differentiation and T-cell priming. Targeted depletion of these Ly6C+ monocytes, while preserving the CCL2-CCR2 recruitment dynamics, redirects monocyte fate toward mature antigen-presenting dendritic cells, preventing resistance, restoring T-cell proliferation, and achieving complete tumor rejection when added to dual ICIs. In contrast, direct CCL2 neutralization attenuates the benefit, illustrating the nuanced therapeutic window for axis modulation (113).

**Figure 4:**
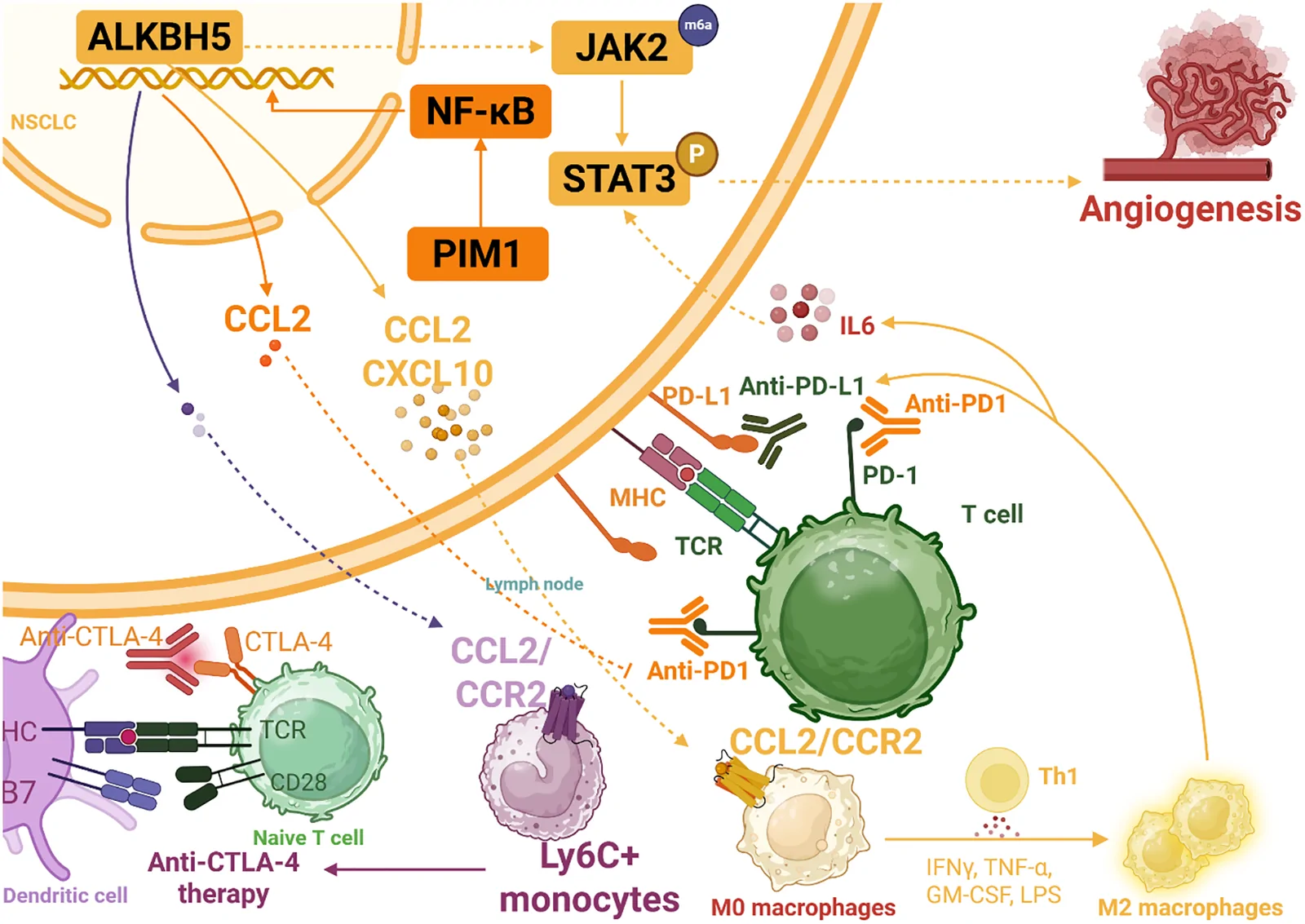
Modulation of the CCL2–CCR2 axis to enhance immune checkpoint blockade in NSCLC. This diagram depicts the multifaceted role of the CCL2–CCR2 signaling axis in shaping the tumor immune microenvironment and its impact on ICI efficacy in non-small cell lung cancer. Tumor-intrinsic pathways, including PIM1-driven NF-κB activation and ALKBH5-mediated m6A demethylation, promote the transcription of CCL2 and related chemokines, facilitating recruitment of CCR2^+^ Ly6C^+^ monocytes and their differentiation into immunosuppressive M2-like TAMs. These TAMs further reinforce immune evasion through IL-6 secretion and activation of the JAK2/STAT3 pathway, leading to increased PD-L1 expression on both tumor and immune cells. The resulting microenvironment suppresses T-cell activation despite antigen presentation via the MHC–TCR complex and co-stimulatory signaling. Therapeutic interventions, including anti-PD-1, anti-PD-L1, and anti-CTLA-4 antibodies, aim to restore T-cell function, while strategies targeting upstream regulators or modulating monocyte recruitment can enhance treatment response. Additionally, approaches such as tumor-treating fields (TTFields) promote immunogenic signaling and chemokine induction, further boosting T-cell infiltration. Collectively, the figure highlights how precise targeting of the CCL2–CCR2 axis can reprogram macrophage dynamics, overcome immunosuppression, and improve the clinical efficacy of ICIs.

As presented in **Figure 4**, these findings establish CCL2-CCR2 targeting as a promising adjunct to enhance ICI efficacy in NSCLC by dismantling macrophage-driven immunosuppression, promoting T-cell infiltration, and enabling durable anti-tumor immunity, paving the way for refined combination strategies in clinical practice.

#### 4.1.3 CCL2-mediated Treg recruitment

The CCL2-CCR2 axis critically orchestrates regulatory T cell (Treg) recruitment to the TME, thereby fostering immunosuppression and resistance to immunotherapies (114, 115). In lung cancer, tumor cell–intrinsic SOX2 expression upregulates CCL2, which engages CCR2 highly expressed on Tregs to drive their preferential accumulation in the peritumoral region. This CCL2-CCR2-mediated Treg enrichment impairs endothelial cell activation and downregulates VCAM-1 on tumor vasculature, excluding CD8+ effector T cells from the tumor core and promoting resistance to checkpoint blockade therapy while leaving systemic T-cell activation intact (116). Although SOX2 also induces CCL7, targeted reduction of CCL2 alone blunts Treg recruitment. Surgical trauma further elevates CCL2, similarly enhancing Treg infiltration and accelerating tumor progression in preclinical models and patients. Anti-GITR therapy selectively depletes GITR-high Tregs enriched in the TME, restoring vascular function, enabling CD8+ T-cell infiltration through a positive feedback loop, and sensitizing SOX2-positive tumors to immunotherapy (117). These mechanisms position disruption of the CCL2-CCR2 axis in Treg recruitment as a promising adjunct to overcome immunotherapy resistance in lung cancer.

### 4.2 Chemotherapeutics and pharmacological compounds

The CCL2-CCR2 axis has been implicated in chemotherapy responses across cancers by modulating immune cell trafficking within the TME, often shifting the balance toward immunosuppression and altered therapeutic efficacy. In lung cancer models, this signaling pathway interacts dynamically with various chemotherapeutic agents and pharmacological compounds, influencing the recruitment of inflammatory cells that shape tumor progression and treatment outcomes (16).

Cisplatin administration in lung cancer elevates reactive oxygen species levels within tumor cells and tissues, which drives the non-enzymatic oxidation of phospholipids and subsequent release of oxidized phospholipids such as oxPAPC. This oxidized phospholipid then acts on macrophages to induce secretion of MCP-1, also known as CCL2, along with LTB-4. Through the MCP-1/CCL2 and LTB4/LTB4R axes, these chemokines promote chemotaxis and infiltration of Ly6C-high monocytes and neutrophils into tumor tissues. The resulting accumulation of myeloid-derived suppressor cells, with monocytes differentiating into tumor-associated macrophages and neutrophils into tumor-associated neutrophils, fosters tumor proliferation, invasion, and metastasis, thereby attenuating the overall antitumor effect of cisplatin despite its initial cytotoxic action (118). In parallel, the tyrosine kinase inhibitor imatinib interferes with this axis by preventing cytokine-induced M2-like polarization of macrophages in response to IL-4 or IL-13. It downregulates surface marker CD206 and M2-associated genes, including Arg1, Mgl2, Mrc1, CDH1, and CCL2, while blocking STAT6 phosphorylation and nuclear translocation. Conditioned medium from M2-like macrophages normally enhances migration of lung cancer cells, yet imatinib restrains this effect. Consequently, in Lewis lung cancer-bearing models, imatinib reduces M2-like macrophage percentages in both tumor and lung tissues after one week, limiting metastatic spread without altering primary tumor growth (119). Similarly, gefitinib exerts comparable effects on the CCL2-CCR2 axis by suppressing IL-13-triggered M2 polarization in macrophages. It diminishes expression of M2 markers such as CD163 and CD206, as well as genes including Mrc1, Fizz1, Arg1, Ym1, and IL-10, through STAT6 pathway inhibition. Tumor-associated macrophages in the microenvironment secrete factors like CCL2 that drive angiogenesis and metastasis, but gefitinib abrogates the tumor-promoting migration induced by M2-conditioned medium on Lewis lung carcinoma cells. This leads to decreased M2-like tumor-associated macrophage accumulation at metastatic sites, highlighting gefitinib’s role in reprogramming the tumor milieu to curb dissemination (120).

When combined with paclitaxel, the imidazoacridinone C-1305 modulates CCL2 levels in lung cancer models such as A549 xenografts. Treatment elevates plasma and tumor CCL2 concentrations, coinciding with shifts in angiogenic signaling, including reduced FGF1, TGF-β, and Ang-4 alongside heightened ERK1/2 and Akt phosphorylation, that influence blood perfusion and vessel organization. Although antitumor activity remains limited in some lung models, the interaction suggests that paclitaxel-inclusive regimens can alter CCL2-CCR2-mediated stromal responses, potentially affecting drug delivery and immune infiltration within the TME (121). As a glucocorticoid, dexamethasone further engages the CCL2-CCR2 axis in lung adenocarcinoma cells expressing high glucocorticoid receptor levels by inducing persistent senescence-associated secretory phenotype components. It sustains overexpression of CCL2, CCL4, CXCL1, and CXCL2, which recruit monocytes, T cells, and natural killer cells to the tumor site. This enhances peripheral blood lymphocyte expansion, tumor infiltration, and cytolytic activity, partly through upregulated NKG2D ligands such as MICA/B, while operating in cytokine contexts like IL-2 and IL-15 that support immune activation even after dexamethasone withdrawal (122).

As demonstrated in **Table 3**, these findings illustrate how the CCL2-CCR2 axis integrates with chemotherapeutics and glucocorticoids to either reinforce immunosuppressive recruitment that limits efficacy or redirect immune responses in lung cancer, offering molecular insights for strategies that target this pathway to improve clinical outcomes.

**Table 3:** Modulation of CCL2 expression by various anticancer drugs in lung cancer and related preclinical models.

Drug	Drug class	CCL2-CCR2 activity	Type of study	Cancer and cell lines	Axis	Drug effect on CCL2	Ref
Cisplatin	Alkylating agent	↑ CCL2	*In vitro* and *in vivo*	Lewis Lung Carcinoma (LLC1), Human breast cancer cell line (MCF-7)	oxPAPC/MDSCs/MCP‐1/CCL2 and LTB4/LTB4R	“Cisplatin induces oxPAPC release mediating MCP‐1/CCL2 and LTB4/LTB4R axis of macrophages to enhance inflammatory cell infiltration into tumour tissues.”	(118)
Dexamethasone	Glucocorticoid	↑ CCL2	*In vitro* and *in vivo*	Lung adenocarcinoma (H1299-GRα Clone 4)	Dex-induced senescence (SASP)	“Extended dexamethasone treatment causes sustained and irreversible increase in CCL2 secretion.”	(122)
Imatinib	Tyrosine kinase inhibitor	↓ CCL2	*In vitro* and *in vivo*	Lewis lung carcinoma (LLC), RAW264.7 macrophages	M2-like polarization/STAT6/CCL2	“Imatinib significantly reduced CCL2 mRNA expression in IL-13-induced M2-like macrophages (BMDMs); contributes to inhibition of M2 polarization.”	(119)
Gefitinib	Tyrosine kinase inhibitor	↓ CCL2	*In vitro* and *in vivo*	Lewis Lung Carcinoma (LLC), RAW 264.7 cells, BMDMs	IL-13/STAT6/M2 polarization/CCL2	“Gefitinib significantly down-regulates IL-13-induced CCL2 expression in macrophages and reduces M2-like polarization.”	(120)
C-1305 + Paclitaxel	Imidazoacridinone + Taxane	↑ CCL2	*In vitro* and *in vivo*	Human lung adenocarcinoma (A549), Human colon carcinoma (HCT116)	Tumour angiogenesis modulation/CCL2/PDGF/FGF1/TGF-β/Ang-4 axis	“Increased plasma CCL2 level after combined treatment; tendency to increase in HCT116 tumour tissue.”	(121)
Docetaxel	Taxane	↑ CCL2	*In vitro*	Human non-small cell lung cancer (A549)	DTX/CCL2/PI3K/AKT/Bcl-2/Bad/caspase-3	“DTX treatment significantly increases CCL2 mRNA and protein expression; CCL2 upregulation contributes to DTX resistance via PI3K/AKT activation and inhibition of apoptosis.”	(123)
Arsenic trioxide (ATO)	Metalloid/chemotherapeutic agent	↓ CCL2	*In vitro* and *in vivo*	Lewis lung carcinoma (LLC), RAW264.7 macrophages; human LUAD clinical samples	Jagged1/Notch1/CCL2/IL-1β/M2 TAM polarization	“ATO inhibits Notch-dependent secretion of CCL2 (and IL-1β) from LUAD cells, thereby blocking M2-like TAM polarization and reducing tumor-promoting effects.”	(124)

#### 4.2.1 Chemoimmunotherapy

Although chemoimmunotherapy has emerged as a promising treatment modality, it is often hindered by severe toxicity of chemotherapeutic agents and the immunosuppressive nature of the TME (125). It interacts with the CCL2-CCR2 chemokine axis to remodel this barrier and augment anti-tumor immunity in lung cancer. An injectable 5-fluorouracil-constituted DNA hydrogel embedded with quercetin (Q-5FDHG), developed through a novel DNA amplification reaction, tackles these issues via progressive enzymatic degradation for continuous release of 5-fluorouracil and quercetin. Quercetin attenuates CCL2 secretion, thereby reducing tumor-associated macrophage recruitment and remodeling the immunosuppressive TME. Simultaneously, 5-fluorouracil inhibits tumor cell proliferation with reduced systemic toxicity by local administration and induces immunogenic cell death to enhance tumor immunogenicity. In orthotopic murine models, Q-5FDHG elicits specific anti-tumor immune responses, resulting in significant inhibition of tumor growth and lung metastasis (126). This unique combination from chemotherapeutic and traditional Chinese medicine sources provides a safe and effective chemoimmunotherapy strategy with great promise for clinical translation in lung cancer.

#### 4.2.2 Chemopreventive

Chemopreventive agents interact with the chemokine axis to improve cancer therapy by disrupting macrophage recruitment and polarization that sustain tumor progression. In lung cancer, the CCL2-CCR2 axis features centrally in this chemopreventive mechanism as lapatinib reduces M2-polarized tumor-associated macrophages and LLC tumor growth through blockade of IL-13-stimulated STAT6 signaling, lowering M2 markers such as CD206, CD163, Arg1, Mrc1, IL-10, Fizz1, and Ym1 while curbing TAM-derived CCL2, IL-4, and IL-13 release. This action reverses *in-vitro* and *in-vivo* polarization, modulates the TME, abolishes IL-13-activated macrophage promotion of LLC motility, and complements lapatinib’s established anti-proliferative effects via EGFR/HER2 and PKM2 pathways across multiple malignancies (127). To wrap up, lapatinib emerges as a promising chemopreventive option for lung cancer by targeting M2 polarization and the CCL2-CCR2 axis.

### 4.3 Epidrugs

**Figure 5:**
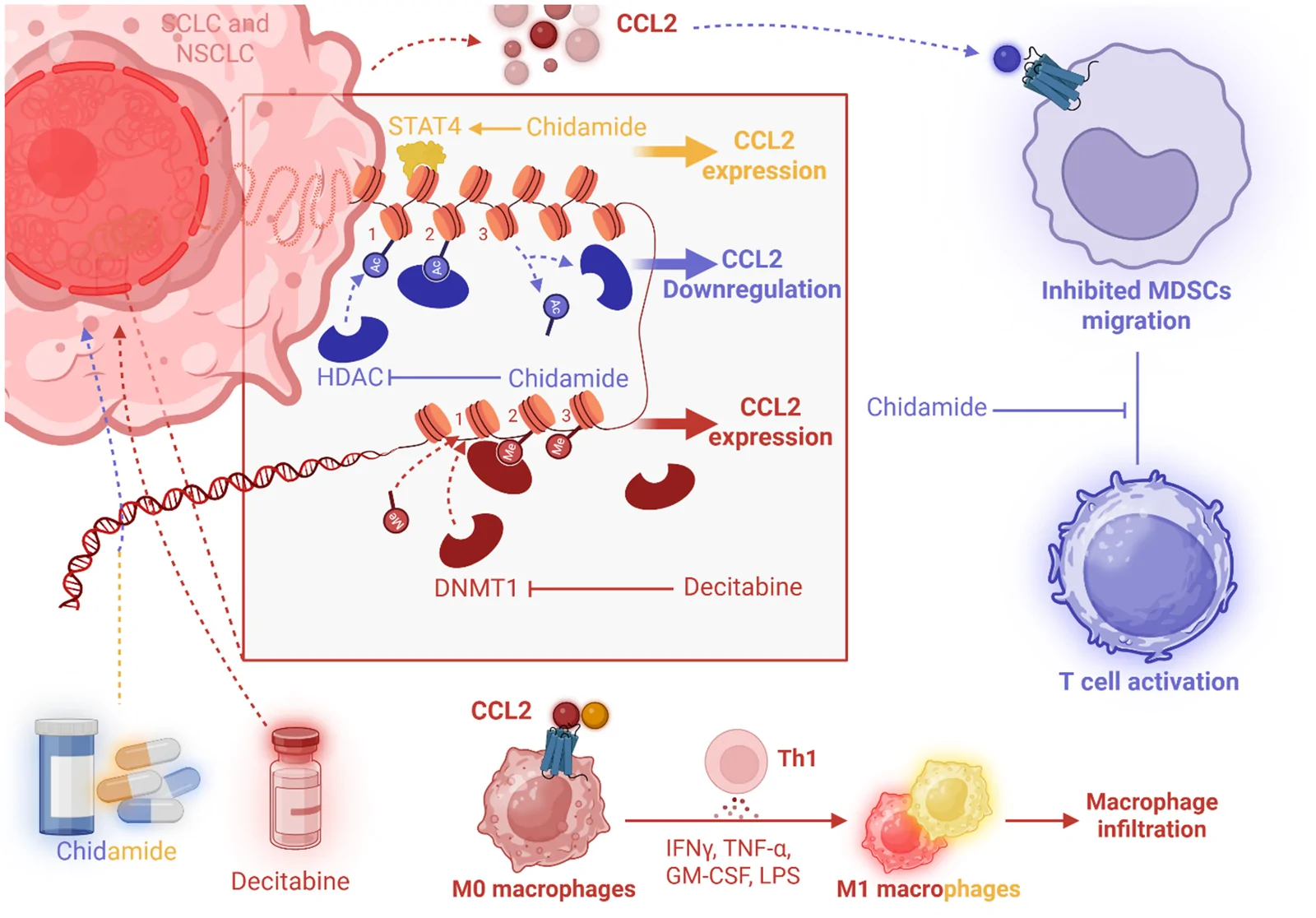
Epigenetic modulation of the CCL2–CCR2 axis as a therapeutic strategy in lung cancer. This schematic illustrates how epigenetic drugs (epidrugs) dynamically regulate the CCL2–CCR2 signaling axis to reshape the tumor immune microenvironment in both SCLC and NSCLC. Histone deacetylase inhibition by chidamide alters chromatin accessibility and transcription-factor binding, resulting in context-dependent regulation of CCL2 expression. In SCLC, chidamide enhances histone acetylation and promotes STAT4-mediated transcription of CCL2, increasing chemokine secretion and recruiting monocytes that differentiate into tumoricidal M1 macrophages, thereby supporting anti-tumor immunity. In contrast, in NSCLC, chidamide suppresses CCL2 production and down-regulates CCR2 expression on monocytic MDSCs, reducing their infiltration and thereby restoring CD8^+^ T-cell activity. Additional epigenetic regulators, including DNMT1 and EZH2, repress CCL2 through DNA methylation and histone methylation; their inhibition by agents such as decitabine reactivates CCL2 expression and enhances macrophage infiltration. Collectively, these mechanisms demonstrate that precise epigenetic reprogramming of the CCL2–CCR2 axis can either stimulate anti-tumor immunity or alleviate immunosuppression, providing a flexible framework for combination therapies in lung cancer.

Epigenetic drugs have gained traction in lung cancer therapy by reversing aberrant histone acetylation, methylation, and DNA methylation patterns that drive tumor growth and immune suppression in malignancies (128, 129). As shown in **Figure 5**, selective histone deacetylase inhibitors (HDACi) such as chidamide, targeting HDAC1/2/3/10, pan-HDACi including valproic acid, EZH2 inhibitors such as EPZ011989, and DNMT inhibitors such as decitabine have shown preclinical efficacy in modulating gene expression within the tumor immune microenvironment.

In SCLC, chidamide directly engages the CCL2-CCR2 axis through epigenetic activation to elicit potent immunomodulatory effects. By elevating histone H3K27 acetylation at the CCL2 promoter and enhancing STAT4 occupancy at conserved binding motifs, chidamide drives STAT4-mediated transcriptional upregulation of CCL2. This mechanism promotes tumor-cell secretion of CCL2, which recruits circulating monocytes and polarizes tumor-associated macrophages toward the antitumor M1 phenotype, thereby counteracting SCLC immune evasion. The resulting immunostimulatory niche synergizes with chidamide’s intrinsic antiproliferative and pro-apoptotic actions on tumor cells. When combined with the multi-targeted tyrosine kinase inhibitor anlotinib, the regimen further neutralizes CCL2-associated angiogenesis, yielding superior tumor control compared with monotherapy in preclinical models (130).

Extending this strategy to NSCLC, chidamide exerts an opposing yet complementary modulation of the CCL2-CCR2 axis to overcome acquired resistance. Gefitinib treatment elevates CCL2 secretion by tumor cells, thereby expanding CCR2-expressing monocytic myeloid-derived suppressor cells (M-MDSCs) that sustain immunosuppression. Chidamide reverses this resistance by suppressing CCL2 production at the tumor site and downregulating CCR2 on M-MDSCs effects linked to increased global histone H3/H4 acetylation that indirectly represses pro-resistance genes. Consequently, the combination reduces M-MDSC infiltration, reactivates CD8+ T-cell function, and restores gefitinib sensitivity, proving more effective than direct CCL2-neutralizing antibodies or CCR2 inhibitors alone (131). Parallel epigenetic approaches targeting repressive marks further refine CCL2-CCR2 regulation in SCLC. DNMT1-mediated CpG methylation and EZH2-driven H3K27me3 silence CCL2 enhancer and promoter regions, restricting macrophage infiltration and maintaining an immunologically “cold” tumor. Decitabine and EPZ011989 relieve this repression, restoring CCL2 expression, enhancing monocyte recruitment via the CCL2-CCR2 axis, and skewing macrophages to the tumoricidal M1 state. Dual inhibition produces additive CCL2 restoration and greater macrophage infiltration than single agents (132).

Collectively, these epidrug strategies demonstrate that precise manipulation of the CCL2-CCR2 axis, whether through activation for macrophage reprogramming or inhibition for MDSC depletion, offers a versatile therapeutic paradigm. By integrating epigenetic modulation with kinase inhibitors or checkpoint blockade, such approaches hold substantial promise for overcoming refractory lung cancer and warrant prioritized clinical evaluation.

### 4.4 Natural compounds

Natural products continue to attract interest in lung cancer management because of their ability to target multiple hallmarks of malignancy while exhibiting favorable safety profiles. Extracts and bioactive constituents from traditional medicinal plants, including those derived from *Salvia miltiorrhiza* Bunge (danshen) and *Magnolia officinalis*, modulate key oncogenic networks and reshape the immunosuppressive TME, offering complementary strategies to conventional therapies (133).

One effective therapeutic avenue centers on disruption of the CCL2-CCR2 axis by natural products and their extractions, which halts the recruitment of M2-like tumor-associated macrophages and breaks the prometastatic crosstalk between cancer cells and the stroma. The aqueous extract of *Salvia miltiorrhiza* Bunge (Sme) potently suppresses lung metastasis in breast cancer models by attenuating CCL2 release from tumor cells, thereby limiting M2 macrophage infiltration through direct blockade of the CCL2-STAT3 signaling axis. This intervention reduces macrophage-driven epithelial-mesenchymal transition and impairs cancer cell motility, demonstrating how natural-product-mediated CCL2-CCR2 inhibition can reprogram the pre-metastatic niche (134). Building on this mechanism, the abietane diterpene dihydroisotanshinone I (DT), a major bioactive constituent of danshen, exerts pronounced effects in both non-small-cell and small-cell lung cancer lines. At 10 μM, DT down-regulates CCL2 secretion from A549 and related lung adenocarcinoma cells while inhibiting STAT3 Tyr705 phosphorylation, dissociating STAT3 from the CCL2 promoter and thereby suppressing macrophage recruitment and tumor cell migration. Conditioned-medium experiments and direct co-culture models confirm that DT interrupts CCL2-CCR2-dependent bidirectional signaling between lung cancer cells and macrophages (RAW 264.7 or THP-1), simultaneously inducing apoptosis and reducing xenograft tumor burden (135).

Complementary natural agents reinforce this paradigm. Honokiol from *Magnolia officinalis* re-educates macrophages from an M2 to an M1 phenotype, down-modulating CCL2 expression and CCR2 surface levels via STAT3/STAT6 inhibition, which markedly decreases pulmonary metastatic nodules in preclinical metastasis models (136). Likewise, the olive-mill-wastewater polyphenol-rich extract A009, sanguinarine, luteolin, and berbamine each suppress CCL2 production or CCL2-dependent monocyte chemotaxis in lung adenocarcinoma and Lewis lung carcinoma systems, further limiting TAM accumulation and cancer-cell invasion (137–140) (**Table 4**).

**Table 4:** Natural compounds targeting the CCL2-CCR2 axis in lung cancer models.

Extraction	Natural compound	Dosage	CCL2-CCR2 activity	Type of study	Cancer and cell lines	Drug effect on CCL2	Ref
Dihydroisotanshinone I (DT)	Salvia miltiorrhiza Bunge (danshen)	10 μM,	↓ CCL2	*In vitro* and *in vivo*	Human lung adenocarcinoma cell line (A549), human lung large cell carcinoma cell line (H460), human small cell lung cancer cell line (H146), human small cell lung cancer cell line (H209), human lung adenocarcinoma cell line (H1650), human normal lung fibroblast (IMR-9), mouse macrophage cell line (RAW 264.7)	• Suppressed the macrophage recruitment ability of lung cancer cells • Induced apoptosis • Inhibited the final tumor volume in a xenograft	(135)
A009 (OMWW polyphenol-rich extract)	Olive mill wastewater	1:5000 to 1:50 dilutions (most active: 1:500, 1:250, 1:100)	↓ CCL2	*In vitro*	Human lung adenocarcinoma cell lines (A549, H1650); also tested on H1299, H1975	• Decreased production of CCL2 in A549 cells • The production of chemokine CXCL12 and CXCR4 receptor were reduced • Interfered with the production of proangiogenic and pro-inflammatory VEGF, CXCL8, and CCL2	(137)
Sanguinarine (SNG)	Sanguinaria canadensis	4 μM	↓ CCL2	*In vitro*	Human lung adenocarcinoma cell line (A549); human acute monocytic leukemia cell line (THP-1)	• SNG-treated exosomes from A549 cells significantly decreased RNA expression levels of CCL2 in THP-1 cells • Inhibited NF-κB pathway in THP-1 macrophages • Reduced macrophage-mediated promotion of A549 proliferation, migration, and invasion • Promoted apoptosis of A549 cells	(138)
Luteolin	flavonoids	1–10 μM (most effective at 10 μM)	↓ CCL2	*In vitro*	Murine macrophage cell line (RAW 264.7), human monocytic leukemia cell line (THP-1), murine Lewis lung carcinoma cell line (LLC)	• Inhibited IL-4-induced STAT6 phosphorylation and M2-like TAM phenotype • Suppressed CCL2 secretion and gene expression from IL-4-stimulated macrophages • Reduced THP-1 monocyte migration in a CCL2-dependent manner • Inhibited migration of LLC cells via CCL2 suppression	(139)
Berbamine	Berberis amurensis	5–90 μM (*in vitro*); 30–90 mg/kg (*in vivo*)	↓ CCL2	*In vitro* and *in vivo*	KRAS-mutant lung adenocarcinoma cell lines (H460, H2122, H23, H358, A549); Lewis lung carcinoma (LLC)	• Reduced M-MDSC infiltration in the TME • Induced cell cycle arrest, senescence, and apoptosis in KRAS-mutant LUAD cells • Suppressed tumor growth in xenograft models	(140)

To sum up, these preclinical findings illustrate the broad therapeutic potential of natural products in dismantling the CCL2-CCR2 axis to restrain lung cancer progression and metastasis. As summarized in the accompanying table, targeted modulation of this pathway by structurally diverse plant-derived compounds provides a rational foundation for future translational studies and combination regimens.

### 4.5 Radiotherapy

Radiotherapy constitutes a primary therapeutic strategy for NSCLC, adeptly shrinking primary tumors via ionizing radiation while offering a viable option for non-surgical candidates. Yet, its influence extends beyond direct cytotoxicity, reshaping the TME in ways that may inadvertently support tumor progression (141).

Subsequent investigations have elucidated the critical role of radiotherapy in activating the CCL2-CCR2 axis through IL-6 upregulation in NSCLC cells. Exposure of tumor lines such as A549 and H157 to irradiation markedly increases IL-6 secretion, which in turn drives the expression of CCL2 and CCL5. This cascade enhances macrophage chemotaxis toward irradiated tumors, as evidenced by *in vitro* migration assays. Genetic knockdown of IL-6 in these cells substantially curtails radiation-induced macrophage recruitment, a finding corroborated in orthotopic xenograft models utilizing luciferase-labeled H157 cells. In these models, tumors derived from IL-6-silenced cells exhibited significantly lower infiltration of both endogenous and exogenously administered macrophages following lung irradiation compared to control tumors (142). To counter these effects, therapeutic strategies targeting the CCL2-CCR2 interaction in conjunction with radiotherapy techniques have shown promise. Mechanistic studies indicate that neutralizing CCL2 and CCL5 effectively diminishes post-irradiation macrophage migration, thereby preventing the accumulation of immunosuppressive tumor-associated macrophages. Clinically, radiotherapy induces a complex remodeling of the tumor milieu, characterized by the release of pro-inflammatory mediators that recruit suppressive cells alongside stimulating anti-tumor immunity. Notably, CCL2 facilitates the infiltration of macrophages with immunosuppressive traits, contributing to treatment failures outside the irradiated field and metastatic spread, while also emerging as a prognostic indicator alongside IL-6 and radiation-associated lymphopenia, which compromises lymphocyte-mediated tumor control (143).

Furthermore, parallel insights from local ablative approaches such as percutaneous radiofrequency ablation in NSCLC underscore the broader relevance of the CCL2-CCR2 axis. Although ablation triggers tumor necrosis and can elicit anti-tumor immune responses through debris release, dendritic cell maturation, and reduced regulatory T cells, incomplete procedures correlate with prompt rises in serum TNF-α, CCL2, and CCL4 levels. These elevations coincide with amplified monocytic myeloid-derived suppressor cell activity, marked by elevated nitric oxide production, thereby fostering relapse and highlighting how targeting CCL2-CCR2 could augment radiotherapy efficacy by curbing chronic inflammatory signals that sustain suppressor-cell function (144). Integrating CCL2-CCR2 blockade with radiotherapy offers a compelling avenue to mitigate unintended pro-tumor effects while harnessing its anti-cancer potential, ultimately improving survival prospects in lung cancer patients.

### 4.6 CCL2-neutralizing antibodies

CCL2-neutralizing antibodies have been explored in various solid tumors to disrupt this chemokine signaling. In preclinical models of breast, prostate, and other cancers, these antibodies effectively reduced tumor cell survival, motility, and metastasis by blocking CCL2-mediated recruitment of immunosuppressive cells and angiogenesis. However, their application in cancer therapy faces notable limitations, including high production and storage costs, as well as the risk of increased metastatic burden upon antibody withdrawal, as observed in orthotopic mammary tumor models (145). In contrast, small-molecule CCR2 antagonists, such as CAS 445479-97-0, offer a more practical approach by directly interrupting the CCL2-CCR2 interaction in lung adenocarcinoma A549 cells. This blockade downregulates MMP-9 expression, thereby suppressing CCL2-driven proliferation, migration, and invasion *in vitro*. Unlike antibodies, CCR2 antagonists avoid rebound progression after cessation and demonstrate improved survival in relevant models. Although the axis exhibits context-dependent dual roles, promoting metastasis while also recruiting anti-tumor gamma-delta T cells, targeting CCR2 holds strong potential for CCR2-positive NSCLC patients by limiting immunosuppressive cell infiltration, Tregs, MDSCs, TAMs, and inhibiting metastatic processes (146).

### 4.7 Nanoparticles

Nanoparticles engage the CCL2-CCR2 axis as a strategic component in cancer therapy, enabling enhanced tumor targeting through chemokine-driven recruitment while mitigating barriers such as inadequate accumulation and swift bloodstream clearance. In the context of lung cancer, macrophage membrane-camouflaged quercetin-loaded hollow bismuth selenide nanoparticles (M@BS-QE NPs) capitalize on CCL2-CCR2 interactions along with immune evasion and active targeting to achieve prolonged circulation and accelerated tumoritropic accumulation relative to bare nanoparticles. Near-infrared laser irradiation prompts quercetin release, which depletes heat shock protein 70, an overexpressed thermoresistance chaperone, to heighten photothermal sensitivity in a synergistic cascade. These nanoparticles further downregulate p-Akt and matrix metalloproteinase-9 signaling to curb extracellular matrix breakdown and suppress metastasis (147). In the end, this biomimetic strategy holds great promise for improving therapeutic efficacy and treatment accuracy with minimal side effects on both primary tumors and metastatic disease.

### 4.8 Promising combination strategies

Building on these advances, rational combination approaches have shown particularly encouraging results (109). Preclinical studies demonstrate that CCR2 blockade synergizes with immune checkpoint inhibitors by reducing immunosuppressive TAM and MDSC infiltration, thereby restoring CD8^+^ T-cell function and enhancing tumor regression in NSCLC models (110, 113). For instance, dual PIM1 and PD-1 inhibition reshapes macrophage polarization, while chidamide combined with gefitinib or anlotinib attenuates MDSC recruitment and overcomes acquired resistance (130, 131). Similarly, pairing CCL2/CCR2 antagonists with chemotherapy or radiotherapy mitigates therapy-induced myeloid cell influx and pre-metastatic niche formation, leading to decreased metastatic burden and improved local control in lung cancer and breast-to-lung metastasis models (118, 142). Early-phase clinical data further support these combinations, with CCR2 antagonists such as PF-04136309 or BMS-813160 showing acceptable safety profiles and enhanced responses when integrated with chemotherapy or nivolumab, underscoring their potential to amplify antitumor immunity while counteracting resistance mechanisms (148, 149). These findings emphasize the value of biomarker-guided, multi-modal strategies that harness the CCL2–CCR2 axis to maximize therapeutic efficacy.

## 5 CCL2-CCR2 and prognostic significance

The CCL2-CCR2 axis has emerged as a pivotal biomarker in prognostic approaches for lung cancer, integrating immune cell trafficking, TME remodeling, and treatment responsiveness to refine risk stratification beyond traditional clinicopathologic features. By governing chemotactic signals that recruit or polarize myeloid and lymphoid populations, this axis modulates antitumor immunity versus immune escape, with expression dynamics and cellular sources yielding subtype- and context-specific survival predictions (150).

In LUAD, CCL2-expressing mast cells (MCs) recruit CCR2+ CTLs displaying activated, tissue-resident memory phenotypes enriched for GZMK, establishing a positive feedback loop that sustains long-term antitumor immunity and correlates with prolonged survival plus superior TKI responses; chemotactic gMCs predominate in ground-glass opacity lesions, whereas solid LUAD reduces MC infiltration and favors angiogenesis-associated subsets (151). Conversely, post-radiotherapy serum CCL2 elevation in NSCLC signals an immunosuppressive TME via TAM accumulation, associating with lymphopenia, higher IL-6, disease recurrence, and inferior outcomes (143). Neutrophil scoring systems further implicate CCL2-driven N2 polarization, upregulating VEGFA, CXCR4, and SPP1 while downregulating FAS, CCL3, and ICAM-1, as a pro-tumor driver, with TNFAIP6 overexpression in tumor cells amplifying this effect and refining prognosis independent of PD-L1 or tumor mutational burden (152). In addition to its prognostic utility, the CCL2-CCR2 axis shows value in distinguishing lung cancer histological subtypes via pleural effusion levels. Patients with advanced lung cancer exhibit elevated CCL2 and CCR2 in pleural effusions compared to parapneumonic effusions, with a moderate positive correlation between the two markers. Levels are significantly higher in LUAD than in LUSC or SCLC, while SCLC shows higher levels than LUSC. Combined detection of CCL2 and CCR2 provides better diagnostic performance than either marker alone or traditional biomarkers such as CEA and CYFRA21-1. This supports its role as a useful supplement to histopathological examination for differentiating LUAD from LUSC, although its value is more limited for distinguishing SCLC from NSCLC and requires integration with other clinical tests (153).

The CCL2-CCR2 axis exhibits opposing prognostic roles across histologies and therapies: high CCL2 predicts unfavorable survival in LUAD yet favorable OS in LUSC, likely reflecting M2 versus M1 TAM skewing; in ICI-treated cohorts, post-treatment CCL2 rises or high change rates of CCL2/CCR1 link to shorter PFS and OS, while hub-gene analyses in LUSC TME confirm CCL2, CCR1, and related mediators as independent poor-outcome predictors. These patterns, summarized in **Table 5**, underscore the axis as a dynamic, actionable prognostic determinant.

**Table 5:** Summary of studies on the CCL2-CCR2 axis in lung cancer prognosis.

Cancer type/setting	Key CCL2-CCR2 observation	Prognostic association	Ref
LUAD (MCs)	CCL2+ MCs recruit CCR2+ CTLs; gMC vs sMC heterogeneity	Favorable survival & TKI response	(151)
NSCLC (post-RT)	Elevated serum CCL2 post-radiotherapy	Poor outcomes, recurrence	(143)
LUAD (TANs)	CCL2 promotes N2 neutrophil polarization via TNFAIP6	Pro-tumor; refines ICI & survival prediction	(152)
LUSC (TME)	CCL2 as TME hub gene	Poor OS	(154)
NSCLC (ICI ± chemo)	Dynamic CCL2/CCR1 changes post-ICI	Poor PFS/OS in high-change subsets	(155)
LUAD vs LUSC	Higher CCL2 in LUAD TME	Unfavorable in LUAD, favorable in LUSC	(156)
NSCLC (ICI)	Serum CCL2 rise in CD24+ low-PD-L1 tumors	Shorter survival post-ICI	(157)

## 6 CCL2-CCR2 in clinical trials

As seen in **Table 6**, the CCL2-CCR2 axis plays a pivotal role in clinical approaches by modulating tumor-associated macrophage recruitment, angiogenesis, and immunosuppression, thereby influencing treatment responses across solid tumors and highlighting its potential as a biomarker and therapeutic target in lung cancer management (150).

**Table 6:** Selected clinical trials of CCL2/CCR2-targeted agents.

Agent	Target	Trial ID(s)	Cancer type(s)	Combination	Status	Key outcomes/notes
BMS-813160 (dual CCR2/5 antagonist)	CCR2/CCR5	NCT04123379	NSCLC, HCC	Neoadjuvant nivolumab ± BMS-813160 (or anti-IL-8)	Active, not recruiting (Phase 2 window-of-opportunity)	Safe and biologically active (increased circulating CCR2/5 ligands, decreased circulating monocytes). Limited major pathologic responses; did not significantly augment the effect of neoadjuvant ICB. Only trial that enrolled patients with lung cancer.
BMS-813160 (dual CCR2/5 antagonist)	CCR2/CCR5	NCT03184870	PDAC, CRC	Chemotherapy (gemcitabine/nab-paclitaxel or FOLFIRI) ± nivolumab	Completed (Phase 1/2)	Acceptable safety; durable responses observed in first-line PDAC cohorts with combination therapy; no clear efficacy advantage demonstrated in CRC cohorts. Data generated exclusively in non-lung malignancies.
BMS-813160 (dual CCR2/5 antagonist)	CCR2/CCR5	NCT03496662	Borderline resectable/locally advanced PDAC	Nivolumab + gemcitabine + nab-paclitaxel	Completed (Phase 1/2)	Tolerable; signals of activity and resectability in selected patients. Data generated exclusively in non-lung malignancies.
BMS-813160 (dual CCR2/5 antagonist)	CCR2/CCR5	NCT03767582	Locally advanced PDAC	Nivolumab ± GVAX + SBRT	Completed (Phase 1/2)	Safety established; exploratory efficacy signals in PDAC. Data generated exclusively in non-lung malignancies.
PF-04136309	CCR2	NCT02732938	Metastatic PDAC	Nab-paclitaxel + gemcitabine	Terminated (Phase 1b/2)	RP2D 500 mg BID established. Notable pulmonary toxicity (≈24% incidence). No efficacy signal above chemotherapy alone. Pulmonary safety signal is of particular relevance when considering future studies in lung cancer. Data generated exclusively in non-lung malignancies.
PF-04136309	CCR2	NCT01413022	Borderline resectable/locally advanced PDAC	FOLFIRINOX	Completed (Phase 1b)	Safe and tolerable at the recommended dose; higher objective response rates and improved local tumor control compared with historical FOLFIRINOX data. Data generated exclusively in non-lung malignancies.
CNTO 888 (Carlumab)	CCL2	NCT00992186	Metastatic castration-resistant prostate cancer	Monotherapy	Completed (Phase 2)	Good tolerability; only transient free CCL2 suppression; no significant PSA or RECIST responses. Illustrates limitations of CCL2 neutralization as monotherapy. Data generated exclusively in non-lung malignancies.

Direct clinical evidence specifically addressing the CCL2-CCR2 axis in lung cancer remains limited. In a large retrospective cohort of patients with refractory advanced NSCLC, anlotinib prolonged both progression-free and overall survival, particularly in those harboring driver mutations such as EGFR T790M. Benefit was accompanied by measurable suppression of serum CCL2, and the magnitude of CCL2 reduction correlated with clinical response, suggesting that dynamic monitoring of circulating CCL2 may serve as a predictive biomarker in the lung-cancer clinic (158). Complementary observations from patients undergoing thoracic radiotherapy or chemoradiation showed that early declines in MCP-1/CCL2 and related mediators preceded radiation-induced pulmonary toxicity, thereby linking axis activity to both antitumor efficacy and normal-tissue injury (159). Among agents that directly engage the axis, the dual CCR2/5 antagonist BMS-813160 has been evaluated in a mixed NSCLC/hepatocellular-carcinoma cohort in combination with nivolumab (NCT04123379); preliminary data indicate acceptable safety and signals of myeloid reprogramming, although lung-cancer-specific efficacy results have not yet been reported in detail (**Table 6**).

### 6.1 Translational insights from other solid tumors

Most interventional experience with selective CCL2 or CCR2 inhibitors has been generated outside lung cancer and must therefore be interpreted as contextual rather than definitive for pulmonary malignancies. In metastatic castration-resistant prostate cancer, the anti-CCL2 monoclonal antibody carlumab produced transient free-CCL2 suppression and was well tolerated, yet yielded no meaningful PSA or RECIST responses, underscoring the limited activity of CCL2 monotherapy (160). In pancreatic ductal adenocarcinoma, the oral CCR2 antagonist PF-04136309 combined with nab-paclitaxel/gemcitabine or FOLFIRINOX established a recommended phase-2 dose of 500 mg twice daily. While monocyte trafficking was measurably reduced, one regimen was associated with notable pulmonary toxicity and neither demonstrated clear superiority over chemotherapy alone (148, 149). Pharmacogenomic analyses of CCL2/CCR2 pathway variants in metastatic colorectal cancer further suggested that host genotype can modulate outcomes under anti-angiogenic therapy in a KRAS-dependent manner (161). Collectively, these data highlight both the biological rationale for targeting the axis and the practical challenges of monotherapy, toxicity (especially pulmonary), and the probable necessity of rational combinations, lessons that are directly informative for ongoing and future lung-cancer trials.

Overall, available clinical observations support continued exploration of CCL2-CCR2 modulation in lung cancer, preferably within biomarker-stratified combination regimens that simultaneously address myeloid immunosuppression, angiogenesis, and treatment-related toxicity.

## 7 Conclusion and perspectives

**Figure 6:**
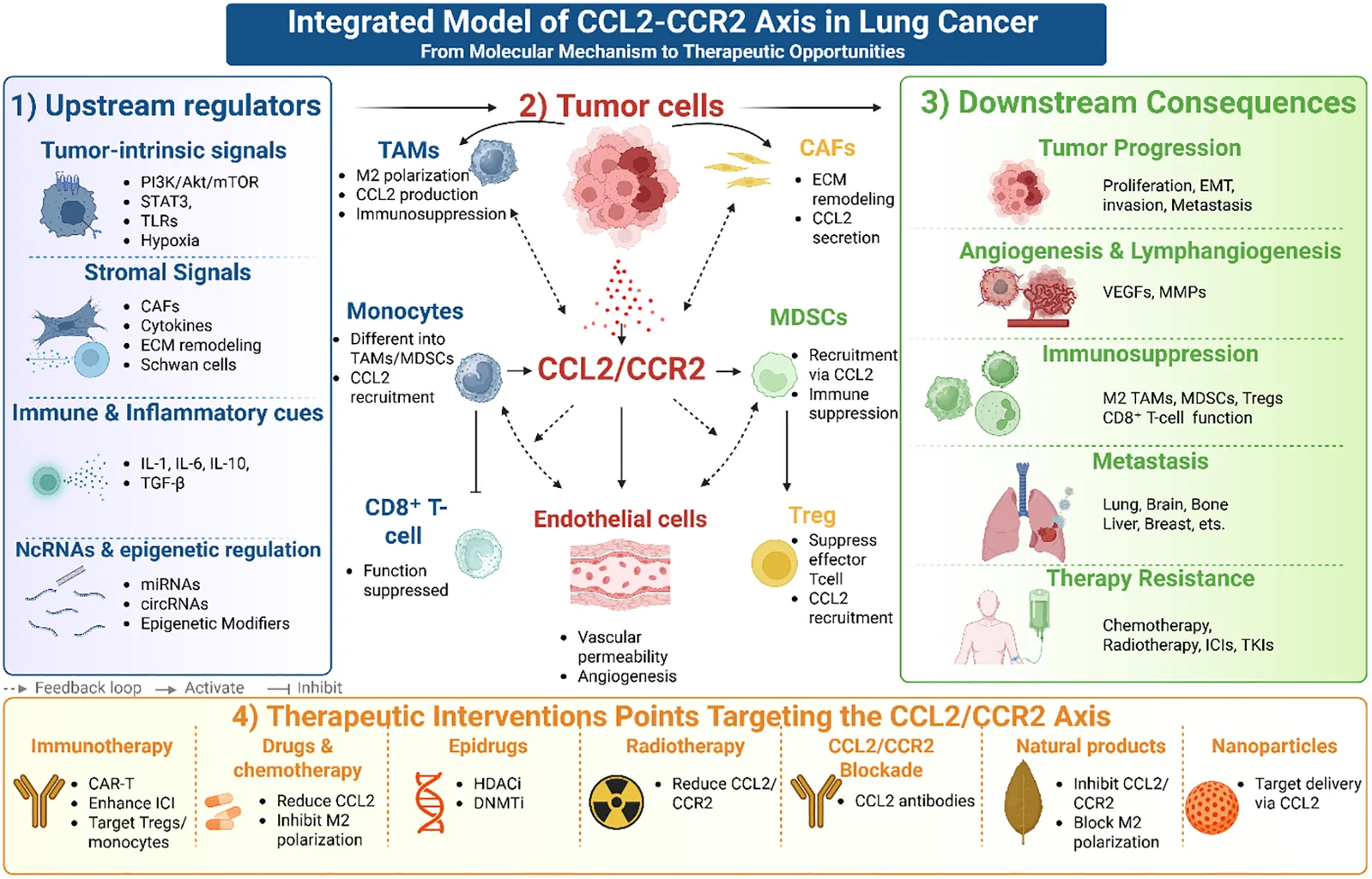
Integrated model of the CCL2–CCR2 axis in lung cancer: from molecular mechanisms to therapeutic opportunities. This schematic summarizes the central role of the CCL2–CCR2 axis as a signaling hub in the lung tumor microenvironment. Diverse upstream regulators (tumor-intrinsic signals, stromal cues, inflammatory mediators, and ncRNA/epigenetic mechanisms) converge to elevate CCL2 production. CCL2 engages CCR2 on monocytes, TAMs, MDSCs, CAFs, endothelial cells, and Tregs, establishing reciprocal feedback loops that drive M2 polarization, immunosuppression, ECM remodeling, angiogenesis, EMT, metastasis, and therapy resistance. The lower panel highlights key therapeutic intervention points, including immunotherapy, conventional drugs and chemotherapy, epidrugs, radiotherapy, direct CCL2/CCR2 blockade, natural products, and nanoparticle-based delivery strategies, that aim to disrupt this axis and restore antitumor immunity.

The CCL2–CCR2 chemokine axis functions as a central signaling hub that integrates tumor-intrinsic, stromal, and myeloid cues to shape the immunosuppressive and pro-metastatic microenvironment of lung cancer. Through recruitment and polarization of M2-like tumor-associated macrophages and myeloid-derived suppressor cells, promotion of epithelial–mesenchymal transition, angiogenesis, and facilitation of distant colonization, the axis contributes to disease progression across molecularly diverse lung cancers. Its regulation involves multiple layers, including PRPS2, CtBP1, neddylation, STAT3, non-coding RNAs, and Toll-like receptor signaling, and extends to crosstalk with cancer-associated fibroblasts, Schwann cells, and therapy-induced host responses. Preclinical studies have generated a compelling biological rationale for therapeutic intervention, demonstrating that disruption of the axis can enhance CAR-T-cell trafficking, improve responses to immune-checkpoint blockade, reprogram tumor-associated macrophages, and impair metastatic niche formation. Small-molecule CCR2 antagonists, neutralizing antibodies, epigenetic modulators, and selected natural compounds have shown encouraging activity in experimental models. Importantly, the axis exhibits clear context- and stage-dependent duality: while it predominantly supports tumor progression in many settings, it can also facilitate antitumor immunity under specific conditions (for example, through recruitment of M1 macrophages or CCR2^+^ cytotoxic T cells). This duality helps explain therapeutic heterogeneity and emphasizes the need for carefully designed, context-aware strategies (**Figure 6**).

Nevertheless, substantial limitations remain. Most mechanistic insights derive from preclinical models that incompletely capture the heterogeneity of human lung cancer, including differences between adenocarcinoma and squamous-cell carcinoma, the influence of smoking history, driver mutations, and comorbidities. Clinical trials of CCL2/CCR2-targeted agents, conducted largely in non-lung malignancies, have shown limited monotherapy efficacy and have raised pulmonary toxicity concerns, highlighting challenges in dosing, patient selection, and combination design. Dynamic changes in CCL2 levels after radiotherapy, chemotherapy, or immunotherapy have not yet been validated as predictive biomarkers, and long-term safety data specific to lung-cancer populations are scarce.

Key unresolved questions include the optimal timing and duration of axis modulation, subtype-specific functional differences, and the identification of reliable stratification biomarkers, tumor or circulating CCL2/CCR2 levels, TAM/MDSC signatures. Interactions with the lung microbiome, metabolic reprogramming, and spatial TME organization, as well as potential resistance mechanisms under sustained targeting, also require clarification. Looking ahead, progress will depend on biomarker-driven clinical trials that incorporate multi-omics profiling, spatial transcriptomics, and single-cell analyses. Priority should be given to rational combinations, CCR2 antagonists with immune-checkpoint inhibitors, CCL2-responsive engineered T cells, or epigenetic agents such as chidamide, together with innovative delivery approaches, tumor-targeted nanoparticles, CCL2 decoys, that may achieve localized effects while minimizing systemic monocyte depletion. Selective harnessing of the axis for immunostimulation, rather than wholesale blockade, represents an additional conceptual avenue.

In summary, the CCL2–CCR2 axis constitutes a biologically compelling candidate target whose therapeutic potential in lung cancer is supported by strong preclinical evidence and early translational observations. Realizing that potential, however, will require rigorous clinical validation through more sophisticated models, refined trial designs, and deeper mechanistic insight. Continued translational efforts that carefully bridge molecular understanding with patient-specific strategies will ultimately determine whether modulation of this network can improve outcomes for patients with advanced lung cancer.
